# Silencing circSERPINE2 restrains mesenchymal stem cell senescence via the YBX3/PCNA/p21 axis

**DOI:** 10.1007/s00018-023-04975-6

**Published:** 2023-10-13

**Authors:** Fenglei Chen, Shan Wang, Chenying Zeng, Su’an Tang, Huimin Gu, Ziming Wang, Jinteng Li, Pei Feng, Yunhui Zhang, Peng Wang, Yanfeng Wu, Huiyong Shen

**Affiliations:** 1 Department of Orthopedics, Eighth Affiliated Hospital of Sun Yat-sen University, Shenzhen, 518033 People’s Republic of China; 2 Center for Biotherapy, Eighth Affiliated Hospital of Sun Yat-sen University, Shenzhen, 518033 People’s Republic of China; 3 Clinical Research Centre, Zhujiang Hospital, Southern Medical University, Guangzhou, 510282 People’s Republic of China

**Keywords:** Mesenchymal stem cells, Cellular senescence, circRNAs, Osteoarthritis

## Abstract

Increasing evidence indicates that circular RNAs (circRNAs) accumulate in aging tissues and nonproliferating cells due to their high stability. However, whether upregulation of circRNA expression mediates stem cell senescence and whether circRNAs can be targeted to alleviate aging-related disorders remain unclear. Here, RNA sequencing analysis of differentially expressed circRNAs in long-term-cultured mesenchymal stem cells (MSCs) revealed that circSERPINE2 expression was significantly increased in late passages. CircSERPINE2 small interfering RNA delayed MSC senescence and rejuvenated MSCs, while circSERPINE2 overexpression had the opposite effect. RNA pulldown followed by mass spectrometry revealed an interaction between circSERPINE2 and YBX3. CircSERPINE2 increased the affinity of YBX3 for ZO-1 through the CCAUC motif, resulting in the sequestration of YBX3 in the cytoplasm, inhibiting the association of YBX3 with the PCNA promoter and eventually affecting p21 ubiquitin-mediated degradation. In addition, our results demonstrated that senescence-related downregulation of EIF4A3 gave rise to circSERPINE2. In vivo, intra-articular injection of si-circSerpine2 restrained native joint-resident MSC senescence and cartilage degeneration in mice with aging-related osteoarthritis. Taken together, our findings provide strong evidence for a regulatory role for the circSERPINE2/YBX3/PCNA/p21 axis in MSC senescence and the therapeutic potential of si-circSERPINE2 in alleviating aging-associated syndromes, such as osteoarthritis.

**Supplementary Information:**

The online version contains supplementary material available at 10.1007/s00018-023-04975-6.

## Introduction

Cellular senescence, which is derived from the Latin word *senex* meaning “old” and was formally described by Hayflick and colleagues in 1961, is a state of permanent cell cycle withdrawal triggered by exposure to numerous endogenous and exogenous stimuli, including genotoxic agents, mitochondrial dysfunction, nutrient deprivation, free radicals, and oncogene activation [1, 2]. During senescence, cells gradually acquire a flattened and enlarged morphology and exhibit activation of a chronic DNA damage response, macromolecular damage and metabolic dysfunction [2]. In addition, they acquire a senescence-associated secretory phenotype (SASP), secreting a series of factors, including proinflammatory cytokines and chemokines, growth regulators, angiogenic factors, and matrix metalloproteinases, which may promote the senescence of the cells themselves and accelerate the senescence of adjacent cells and tissues [3]. Senescent cells are a double-edged sword in human physiology and pathology [4]. Cellular senescence is recognized as an evolutionarily conserved stress response with indispensable and beneficial functions, such as preventing the propagation of damaged cells and contributing to tissue repair and tumor suppression [5, 6]. On the other hand, senescent cells are thought to contribute to organismal aging by accumulating in degenerative tissues, thus promoting a proinflammatory microenvironment and hindering the generation of new cells [7, 8].

Stem cell senescence leads to stem cell exhaustion and hence results in physiological and pathological aging [9]. For example, mesenchymal stem cell senescence has been suggested to cause aging-related tissue degeneration [10]. MSCs are adult multipotent stem cells that can differentiate into osteoblasts, chondrocytes, and adipocytes, which contribute to mesodermal tissue repair [11]. MSC population exhaustion is observed in patients with Hutchinson-Gilford progeria syndrome (HGPS) and Werner syndrome (WS), which are characterized by functional degeneration of mesodermal tissue, such as osteoarthritis (OA), osteoporosis and atherosclerosis [12–14]. Accumulation of senescent MSCs is found in the degenerated tissue of aging-related OA and aging-related osteoporosis, which plays an important role in their pathophysiology [15]. Hence, the potential of strategies targeting MSCs to alleviate or reverse tissue degeneration and aging phenotypes through either elimination of senescent cells by chemotherapeutics or genome reprogramming in vivo has recently attracted much attention [16, 17]. However, stem cell senescence is a complicated physiological and pathological phenomenon caused by multiple factors and mechanisms. Therefore, it is necessary to try to determine the intricate mechanism of MSC senescence and identify an efficient target for ameliorating tissue degeneration.

Recently, numerous studies have demonstrated that the levels of circular RNAs are high in nonproliferating cells but are reduced in cancer cells [18, 19]. CircRNAs are a large category of noncoding RNAs derived from noncanonical splicing events known as backsplicing. Due to the lack of polyadenylation (poly (A)) and capping, circRNAs possess a covalent circular ring structure with a downstream splice-donor site linked to an upstream splice-acceptor site, providing them with high stability against exonuclease-mediated degradation. Although backsplicing is less efficient than linear splicing, circRNAs can accumulate in cells that cease to divide [20]. Instead of being considered to be ‘junk’ generated by aberrant splicing events, circRNAs have been reported to participate in cellular processes, including senescence, proliferation and differentiation, by sponging miRNA, binding proteins or translating peptides in recent years [21, 22]. Currently, the interactions between circRNAs and proteins are receiving more attention than the most well-known function of circRNAs, i.e., their miRNA sponging function. Some interactions between circRNAs and proteins have been shown to play a pivotal role in regulating cellular senescence [21, 23]. Focusing on the interactions between circRNAs and RNA-binding proteins might provide novel insight into cellular senescence.

Y-box binding protein 3 (YBX3) belongs to the conserved Y-box family of multifunctional nucleic acid binding proteins and has been proposed to play a general role in promoting proliferation [24]. YBX3 can bind to promoters, full-length mRNA or even short RNA sequences with specific motifs [25]. Several studies have demonstrated that circRNAs can interact with proteins from this family and affect their expression or subcellular location, resulting in the impairment of cell proliferation or tissue regeneration [21, 26]. To date, whether the interaction between circRNAs and YBX3 is involved in the regulation of the aging process in human MSCs remains largely unknown.

Here, we postulate that during MSC senescence development, circRNAs accumulate and aggravate senescence, thereby accelerating the development of aging-related diseases. We performed RNA sequencing (RNA-seq) of early-passage, middle-passage and late-passage MSCs in our previous work [27]. We identified that the circRNA SERPINE2 accumulated during MSC senescence and promoted MSC senescence through sequestration of YBX3 in the cytoplasm, thus inhibiting its transcriptional regulation of PCNA to inhibit p21 degradation. Of crucial importance, knockdown of circSerpine2 alleviated cellular senescence, significantly diminishing cartilage degeneration and osteophyte formation in mouse OA. Taken together, our findings reveal the role of circSERPINE2 in MSC senescence for the first time and show that the circSERPINE2/YBX3/PCNA/p21 axis plays a key role in the effect of circSERPINE2. It is hoped that circSERPINE2 will become an effective target for treating aging-associated disorders such as OA.

## Results

### CircSERPINE2 accumulates during MSC senescence

**Fig. 1:**
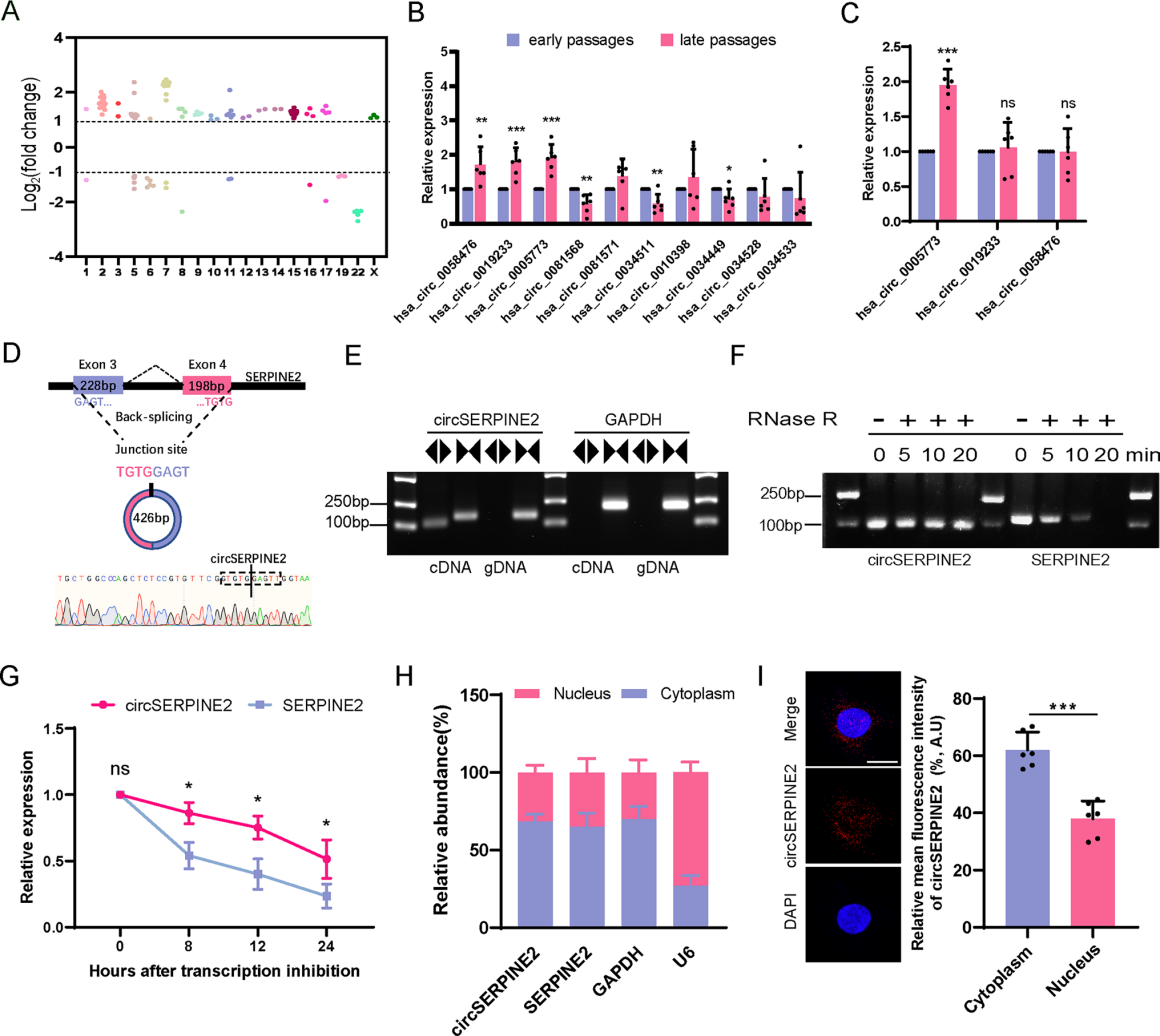
CircSERPINE2 accumulated during MSC senescence.** A** Scatter plot of the RNA-seq data showing the DE circRNAs between P4 MSCs and P12 MSCs, which showed the same trend as the DE circRNAs between P4 MSCs and P6 MSCs (fold change ≥ 2, *Q* < 0.001). X axis, position on the chromosome. **B** Expression levels of the indicated circRNAs, which were chosen from the ten most abundant and upregulated circRNAs in P12 MSCs relative to P4 MSCs. The data are presented as the mean ± SD; *n* = 6. *P < 0.05, **P < 0.01, ***P < 0.001 (two-tailed *t test*). **C** RT–qPCR analysis of hsa_circ_0005773, hsa_circ_0019233 and hsa_circ_0058476 expression in H_2_O_2_-treated MSCs. The data are presented as the mean ± SD; *n* = 6 biological replicates. ***P < 0.01 (two-tailed *t* test). **D** Schematic of exons 3 and 4 of SERPINE2 mRNA, which is located on human chromosome 2 and gives rise to the 426-nucleotide-long circSERPINE2, and details of the sequence junction. **E** The presence of circSERPINE2 was validated by RT–PCR. Divergent primers amplified circSERPINE2 from cDNA but not from genomic DNA. GAPDH was used as a negative control. **F** Comparison of circSERPINE2 and SERPINE2 mRNA expression levels by RNase R digestion analysis at different time points. **G** RT–qPCR was used to determine the levels of circSERPINE2 and its linear counterpart SERPINE2 in MSCs treated with actinomycin D at the indicated time points. The data are presented as the mean ± SD; *n* = 3 biological replicates. *P < 0.05 (two-tailed *t test*). **H** Relative levels of circSERPINE2 in the nuclear and cytoplasmic compartments of MSCs; the quality of the fractionation was assessed by monitoring the levels of a predominantly cytoplasmic transcript (GAPDH mRNA) and a predominantly nuclear mRNA (U6). The data are presented as the mean ± SD; *n* = 3 biological replicates. **I** FISH was used to visualize the subcellular distribution of circSERPINE2. The nuclei were stained with DAPI. Scale bar = 20 µm. The relative mean intensity of circSERPINE2 is presented as the mean ± SD; *n* = 6 biological replicates. ***P < 0.001 (two-tailed *t* test)

We performed whole-genome RNA-seq of early-passage MSCs (P4), middle-passage MSCs (P6) and late-passage MSCs (P12) in our previous work [27]. The results showed that 229 circRNAs were differentially expressed (DE) in P12 MSCs compared with P4 MSCs (fold change |log2 [P12/P4]|≥ 1, Q value [P12/P4] < 0.001), and the same expression trends were observed for P6 MSCs compared with P4 MSCs. Among the 229 DE circRNAs, 202 were upregulated in late-passage MSCs, and only 17 were downregulated (Fig. 1A). Ten circRNAs that were abundant and highly expressed in late-passage MSCs compared with early-passage MSCs were chosen. CircRNA expression levels were determined by real-time quantitative polymerase chain reaction (RT–qPCR) with divergent primers to distinguish circRNAs from their cognate mRNAs. The results showed that the levels of three circRNAs (hsa_circ_0058476, hsa_circ_0019233, and hsa_circ_0005773) were significantly increased in late-passage MSCs, exhibiting the same expression trends as RNA-seq (Fig. 1B). However, only hsa_circ_0005773 was also upregulated in H_2_O_2_-induced senescent MSCs (Fig. 1C). In addition, knocking down hsa_circ_0058476 or hsa_circ_0019233 exerted no effect on MSC senescence (Fig. S1A, B). Therefore, we selected hsa_circ_0005773, whose levels steadily increased in senescent MSCs, for further investigation.

Subsequently, we performed Sanger sequencing analysis of the PCR products of hsa_circ_0005773. The sequence of hsa_circ_0005773 was in accordance with that shown in circBase (http://circbase.org/), and the presence of its backsplicing junction site was verified, consistent with our previous result [27]. Hsa_circ_0005773 is a novel circRNA generated by the circulation of exons 3 and 4 of SERPINE2 pre-mRNA and has a length of 426 nucleotides; thus, we renamed it circSERPINE2 (Fig. 1D). Divergent primers amplified circSERPINE2 from cDNA but not gDNA, indicating its circular structure (Fig. 1E). An RNase R digestion experiment and actinomycin D assay demonstrated that circSERPINE2 was more resistant to RNase R and had a longer half-life than its linear transcript (Fig. 1F and G). These analyses provide powerful evidence of the *bona fide* circRNA structure of circSERPINE2.

Finally, since recent studies have suggested that the function of circRNAs is tightly associated with their subcellular localization [28], we performed RT–qPCR analysis of circSERPINE2 expression in nuclear/cytoplasmic fractions from MSCs. The results indicated that circSERPINE2 was mainly localized in the cytoplasm, with low levels found in the nucleus (Fig. 1H). FISH directly demonstrated that more circSERPINE2 was located in the cytoplasm than in the nucleus (Fig. 1I). Taken together, these data suggest that circSERPINE2 is a novel circRNA that accumulates in senescent MSCs.

### CircSERPINE2 impacts MSC senescence

**Fig. 2:**
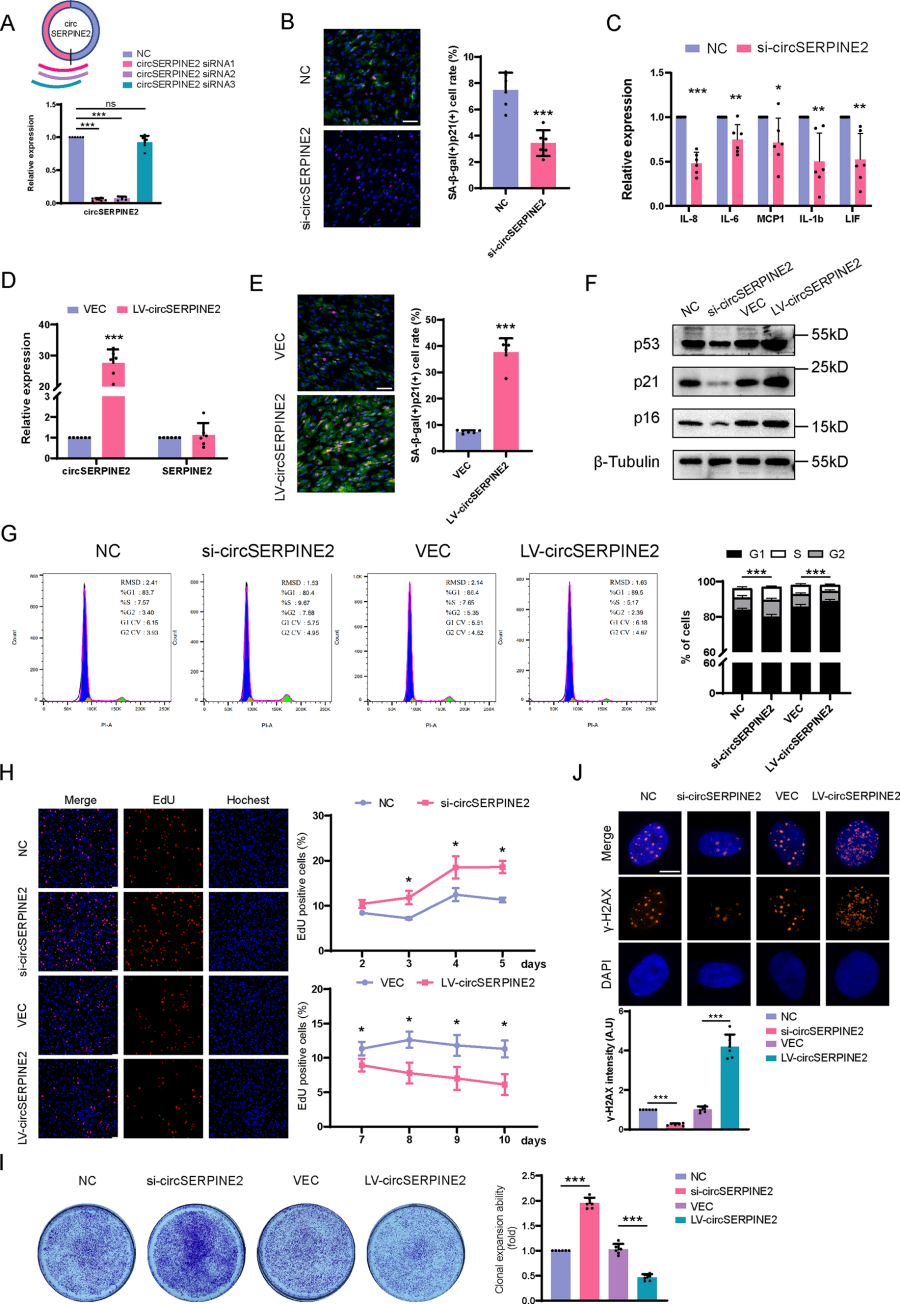
CircSERPINE2 impacts MSC senescence. **A** Schematic of the 3 siRNAs designed to target the circSERPINE2 junction site. RT–qPCR analysis of the levels of circSERPINE2 in MSCs. RNA levels were normalized to the level of GAPDH mRNA. The data are presented as the mean ± SD; *n* = 6 biological replicates. ***P < 0.001 (two-tailed *t* test). **B** β-gal staining and p21 immunofluorescence staining of MSCs transfected with NC siRNA or si-circSERPINE2 for 48 h. Scale bar = 50 µm. The SA-β-gal- and p21-positive cell rates were determined by ImageJ. The data are presented as the mean ± SD; *n* = 6 biological replicates. ***P < 0.001 (two-tailed *t test*). **C** RT–qPCR analysis of the mRNA expression of SASPs in MSCs after silencing circSERPINE2. The data are presented as the mean ± SD; *n* = 6 biological replicates. *P < 0.05, ***P < 0.01, ***P < 0.001 (two-tailed *t* test). **D** MSCs were transfected with LV, and after 7 d of transfection, the level of circSERPINE2 was measured by RT–qPCR and normalized to the level of GAPDH mRNA. The data are presented as the mean ± SD; *n* = 6 biological replicates. ***P < 0.001 (two-tailed *t* test). **E** β-gal staining and p21 immunofluorescence staining of MSCs transfected with LV vector or LV-circSERPINE2 for 7 d. Scale bar = 50 µm. The SA-β-gal- and p21-positive cell rates were determined by ImageJ. The data are presented as the mean ± SD; *n* = 6 biological replicates. ***P < 0.001 (two-tailed *t* test). **F** western blot analysis of p53, p21 and p16 protein expression in MSCs with silencing or overexpression of circSERPINE2. β-Tubulin was used as the loading control. *n* = 6 biological replicates. **G** Flow cytometry of MSCs transfected with NC siRNA, si-circSERPINE2, LV vector, or LV-circSERPINE2. The ratio of (S + G2) phases was analyzed by FlowJo. *n* = 6 biological replicates. ***P < 0.001 (two-tailed *t* test). **H** EdU staining of MSCs transfected with NC siRNA, si-circSERPINE2, LV vector, or LV-circSERPINE2 for different durations. Scale bar = 100 µm. The EdU-positive cell rate was determined by ImageJ. The data are presented as the mean ± SD; *n* = 3 biological replicates for each timepoint. *P < 0.05 (two-tailed *t* test). **I** Analysis of the clonal expansion of MSCs transduced with NC siRNA, si-circSERPINE2, LV vector or LV-circSERPINE2. Clonal expansion ability was analyzed by ImageJ. The data are presented as the mean ± SD; *n* = 6 biological replicates. ***P < 0.001 (two-tailed *t* test). **J** Immunofluorescence analysis of γ-H2AX in MSCs transfected with NC siRNA, si-circSERPINE2, vector LV or LV-circSERPINE2. The γ-H2AX intensity was determined by ImageJ. The data are presented as the mean ± SD; *n* = 6 biological replicates. ***P < 0.001 (two-tailed *t* test). γ-H2AX (orange) and DAPI (blue). Scale bar = 10 µm

To investigate the biological function of circSERPINE2 in MSC senescence, we designed several small interfering (si)RNAs targeting the junction site of circSERPINE2. Transfection of both siRNA1 and siRNA2 significantly reduced circSERPINE2 levels in MSCs; however, siRNA3 did not significantly affect circSERPINE2 levels (Fig. 2A). Thus, we chose siRNA1 (si-circSERPINE2), which exhibited the highest knockdown efficiency, for subsequent experiments. However, the knockdown of circSERPINE2 was accompanied by downregulation of the expression of the linear transcript SERPINE2 (fig. S1C). SERPINE2 belongs to the serpin family, which inhibits serine proteases and plays an important role in angiogenesis and tumor metastasis [29–31]. Our results showed that neither knockdown nor overexpression of SERPINE2 impacted MSC senescence (Fig. S1D-G), supporting further studies on the role of circSERPINE2 in MSC senescence.

SA-β-gal activity has long been recognized as a prominent hallmark of cellular senescence [3]. Recent work has proposed that costaining SA-β-gal and other markers of senescence is a more specific method for recognizing senescent cells [2]. After circSERPINE2 levels in MSCs were reduced by transfection of si-circSERPINE2, the percentage of SA-β-gal- and p21-positive cells was significantly reduced (Fig. 2B). In addition, knockdown of circSERPINE2 inhibited the expression of some SASPs, such as IL-6, IL-8, IL-1b, MCP1 and LIF (Fig. 2C). Then, we constructed a lentivirus (LV) to specifically express circSERPINE2 (LV-circSERPINE2) and assessed whether overexpression of circSERPINE2 aggravates MSC senescence. Overexpression of circSERPINE2 did not affect the level of SERPINE2, indicating its specificity (Fig. 2D). An increased percentage of SA-β-gal- and p21-positive cells was observed in MSCs transfected with LV-circSERPINE2 (Fig. 2E). In addition, senescent cells are characterized by permanent withdrawal from the cell cycle [32]. Thus, we transfected MSCs with either si-circSERPINE2 or LV-circSERPINE2 and examined the expression of the cell cycle arrest-related proteins p53, p21, and p16 by western blot analysis. The results showed that knockdown of circSERPINE2 dramatically decreased p21 protein expression, while the expression of p53 and p16 was modestly downregulated. Consistent with this finding, the protein expression of p53, p21, and p16 was upregulated after LV-circSERPINE2 transfection (Fig. 2F and Fig. S1H). In addition, flow cytometry showed that circSERPINE2 knockdown increased the S- and G2-phase ratios, while overexpression of circSERPINE2 promoted MSC cell cycle arrest (Fig. 2G). CircSERPINE2 also impaired the proliferative potential of MSCs, another prominent feature of cellular senescence. The percentage of EdU-positive cells was higher after transfection of si-circSERPINE2, while LV-circSERPINE2 had the opposite effect (Fig. 2H). Consistent with the EdU staining results, knocking down circSERPINE2 increased the clonal expansion ability, whereas overexpression of circSERPINE2 impaired MSC clonal expansion (Fig. 2I). Furthermore, the immunofluorescence intensity of γ-H2AX, a marker of DNA damage that usually accompanies cellular senescence, declined after silencing circSERPINE2, in contrast to that after circSERPINE2 overexpression (Fig. 2J). In summary, our data demonstrate that circSERPINE2 positively regulates MSC senescence in vitro.

### CircSERPINE2 binds and sequesters YBX3 in the cytoplasm

**Fig. 3:**
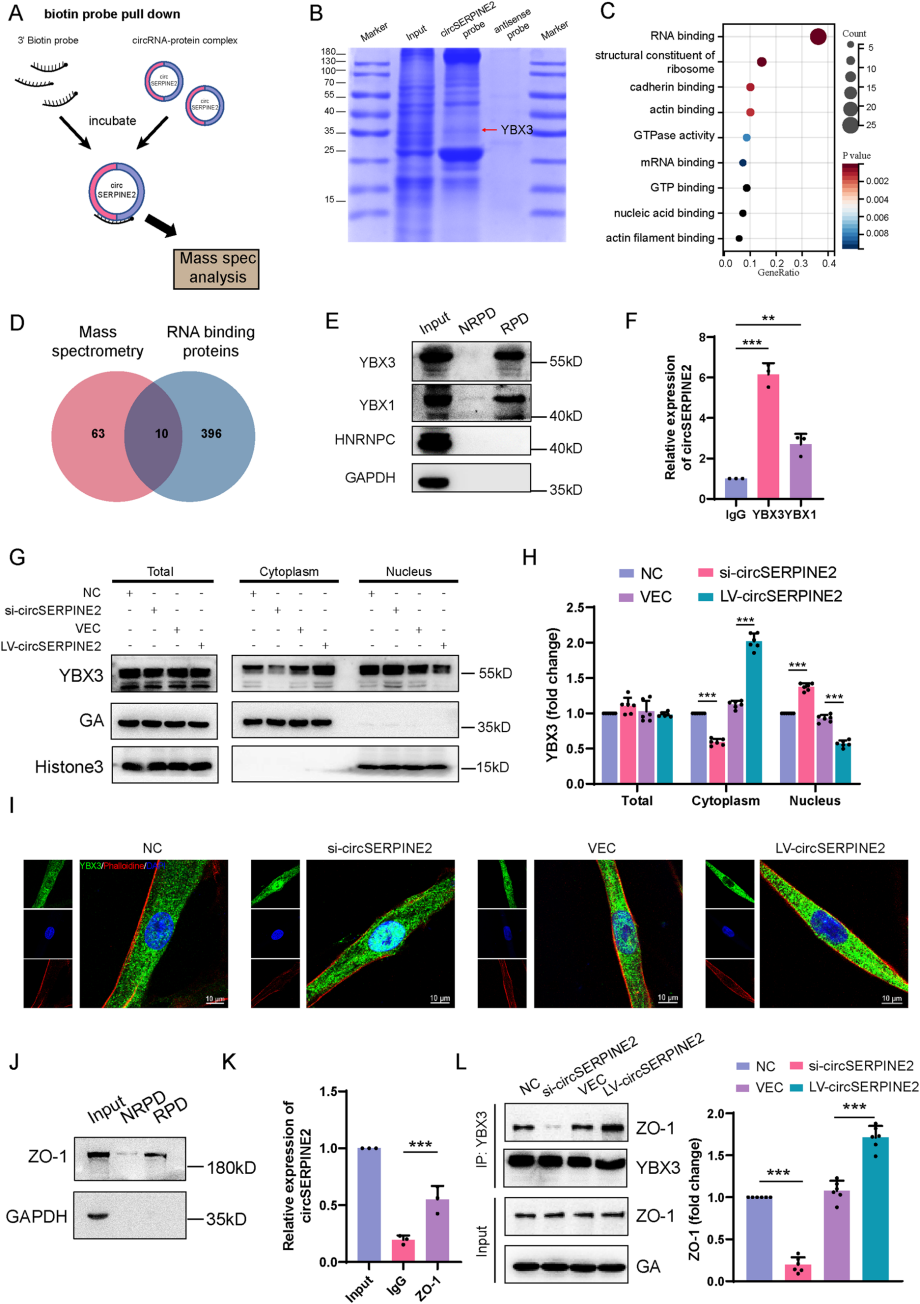
CircSERPINE2 retained YBX3 in the cytoplasm. **A** Schematic diagram of the biotin probe targeting the junction site of circSERPINE2. **B** Coomassie brilliant blue staining followed by mass spectrometry was used to identify the circSERPINE2-protein complex pulled down by the circSERPINE2 probe in protein extracts from MSCs. **C**, Enriched GO categories for the proteins pulled down by circSERPINE2. **D** RNA binding proteins were identified with RBPDB (http://rbpdb.ccbr.utoronto.ca/). **E** YBX3, YBX1 and HNRNPC levels in MSCs were measured by western blot analysis after pulldown with a circSERPINE2 probe or antisense probe. *n* = 3 independent experiments. NRPD antisense probe pulldown, RPD circSERPINE2 probe pulldown. **F** RIP and RT–qPCR were performed to evaluate the interaction between YBX3 and circSERPINE2 in MSCs. The data are presented as the mean ± SD; *n* = 3 biological replicates. **P < 0.01, ***P < 0.001 (two-tailed *t* test). **G** and **H** YBX3 protein levels in total protein, cytoplasmic protein and nucleic protein extracts from MSCs transfected with siRNA or LV. GAPDH was used as a cytoplasmic control, while H3 was used as a nucleic control. The data are presented as the mean ± SD; *n* = 6 biological replicates. ***P < 0.001 (two-tailed *t* test). **I** Immunofluorescence staining of YXB3 (green), DAPI staining (blue) and phalloidin staining (red) in MSCs with knockdown or overexpression of circSERPINE2. *n* = 6 biological replicates. Scale bar = 10 µm. **J** ZO-1 levels in MSCs were measured by western blot analysis after pulldown with a circSERPINE2 probe or antisense probe. *n* = 3 independent experiments. **K** RIP and RT–qPCR were performed to evaluate the interaction between ZO-1 and circSERPINE2 in MSCs. The data are presented as the mean ± SD; *n* = 3 biological replicates. ***P < 0.001 (two-tailed *t* test). **L** Cell lysates were immunoprecipitated with an antibody against YBX3 and analyzed by immunoblotting with an anti-ZO1 antibody or anti-YBX3 antibody. The data are presented as the mean ± SD; *n* = 6 biological replicates. ***P < 0.001 (two-tailed *t* test)

To further determine how circSERPINE2 accelerates MSC senescence, we performed RNA pulldown assays of circSERPINE2 using biotinylated probes targeting the circSERPINE2 backsplice sequence, and the immunoprecipitates were subjected to mass spectrometry analysis (Fig. 3A and B and Supplementary file2). Gene Ontology (GO) enrichment analysis showed that the molecular function of the precipitates was mainly associated with RNA binding (Fig. 3C). We found that 10 of the 74 proteins pulled down by the circSERPINE2 probes were RNA-binding proteins (RBPs) according to RBPDB (http://rbpdb.ccbr.utoronto.ca), and three RBPs that exhibited maximum peptide counts were selected for further investigation (Fig. 3D and Supplementary file2). RNA pulldown assays showed that both YBX3 and YBX1 bound circSERPINE2, while HNRNPC did not (Fig. 3E). Moreover, RIP confirmed that endogenous YBX3/YBX1 interacted with circSERPINE2 in MSCs, further verifying the specificity of the binding (Fig. 3F and Fig. S2A). Previous reports have shown that the Y-box binding protein family comprises multifunctional transcription factors that regulate genes related to proliferation [33, 34]. The interaction between circRNAs and proteins mainly affects the abundance or subcellular localization of proteins [35]. Thus, we investigated the change in the expression and cellular localization of YBX3. The results showed that transfection with neither si-circSERPINE2 nor LV-circSERPINE2 affected the protein levels of total YBX3 or YBX1 (Fig. 3G and Fig. S2B). Then, we explored whether the interactions between circSERPINE2 and YBX3/YBX1 influence the cytoplasmic and nuclear distribution of YBX3/YBX1. Knockdown of circSERPINE2 significantly increased the transport of the YBX3 protein into the nucleus but decreased the level of this protein in the cytoplasm, while ectopic expression of circSERPINE2 promoted the cytoplasmic distribution of YBX3 (Fig. 3G and H), which was not observed for YBX1 (Fig. S2C-D). Immunofluorescence further confirmed the change in YBX3 distribution (Fig. 3I). In line with this finding, YBX3 was more abundant in the nuclei of young MSCs than in the nuclei of senescent MSCs (Fig. S2E, F). Therefore, YBX3 was chosen for subsequent studies. To determine how circSERPINE2 retains YXB3 in the cytoplasm, we investigated the binding between YBX3 and ZO-1, a protein known to restrict YBX3 in the cytoplasm [36]. Both the RNA pulldown assay and RIP assay demonstrated that there was also binding between circSERPINE2 and ZO-1 (Fig. 3J, K). Therefore, we hypothesized that circSERPINE2 might serve as a scaffold to enhance the interaction between YBX3 and ZO-1, thus sequestering YBX3 in the cytoplasm. We performed Co-IP to determine the change in the interaction between YBX3 and ZO-1. Alterations in the expression of circSERPINE2 did not affect ZO-1 expression but significantly affected the interaction between YBX3 and ZO-1 (Fig. 3L). Colocalization analysis by immunofluorescence further confirmed that circSERPINE2 could bind and sequester YBX3 in the cytoplasm by cementing the interaction between YBX3 and ZO-1 (Fig. S2G).

### CircSERPINE2 binds YBX3 through the CCAUC motif

**Fig. 4:**
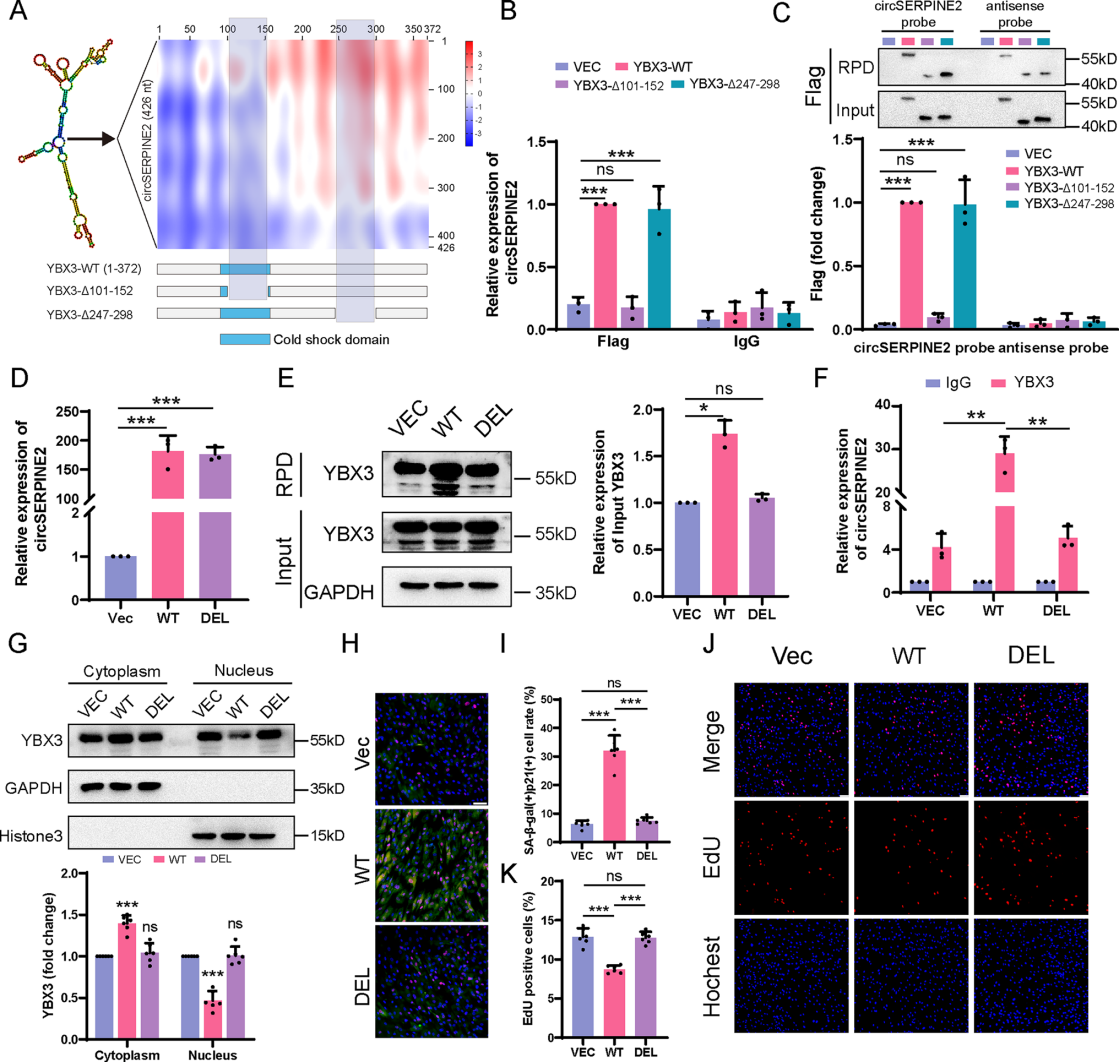
CircSERPINE2 interacted with YBX3 through a specific motif. **A** Prediction of the circSERPINE2-YBX3 interaction by using the catRAPID algorithm and schematic of the YBX3 mutant lacking the 101–152 or 247–298 regions. **B** Relative enrichment of circSERPINE2 in Flag immunoprecipitates (RIP) from HEK293T cells transfected with vector, YBX3-WT, YBX3-Δ101-152 or YBX3-Δ247-298, as measured by RT–qPCR. All data are presented as the mean ± SD; *n* = 3 biological replicates. ***P < 0.001 (one-way ANOVA). **C** Western blot analysis of RNA pulldown precipitates from HEK293T cells transfected with vector, YBX3-WT, YBX3-Δ101-152 or YBX3-Δ247-298. The expression levels of Flag were determined by ImageJ. RPD, RNA pulldown. The data are presented as the mean ± SD; *n* = 3 biological replicates. ***P < 0.001 (one-way ANOVA). **D** RT–qPCR analysis of the transfection efficiency of the different plasmids in HEK293T cells. The data are presented as the mean ± SD; *n* = 3 biological replicates. ***P < 0.001 (one-way ANOVA). **E** western blot analysis of RNA pulldown precipitates from HEK293T cells transfected with vector, circSERPINE2-WT or circSERPINE2-DEL. The expression levels of YBX3 were determined by ImageJ. The data are presented as the mean ± SD; *n* = 3 biological replicates. *P < 0.05 (one-way ANOVA). **F** Relative enrichment of circSERPINE2 in YBX3 immunoprecipitates (RIP) from HEK293T cells transfected with vector, circSERPINE2-WT or circSERPINE2-DEL, as measured by RT–qPCR. All data are presented as the mean ± SD; *n* = 3 biological replicates. **P < 0.01 (one-way ANOVA). **G** Nuclear/cytoplasmic localization of YBX3 in HEK293T cells, as determined by western blotting, after vector, circSERPINE2-WT or circSERPINE2-DEL transduction. The purity of the subcellular fractions was assessed by western blotting for GAPDH (cytoplasm) and Histone3 (nucleus). The data are presented as the mean ± SD; *n* = 6 biological replicates. ***P < 0.001 versus the vector group using one-way ANOVA. **H** and** I** β-Gal staining and p21 immunofluorescence staining of MSCs transduced with vector, circSERPINE2-WT or circSERPINE2-DEL for 7 d. The positive cell rates were determined by ImageJ. The data are presented as the mean ± SD; *n* = 6 biological replicates. ***P < 0.001 versus the vector group (one-way ANOVA). **J** and** K** EdU staining of MSCs transduced with vector, circSERPINE2-WT or circSERPINE2-DEL for 7 d. The positive cell rates were determined by ImageJ. The data are presented as the mean ± SD; *n* = 6 biological replicates. ***P < 0.001 versus the vector group (one-way ANOVA)

Y-box binding proteins have been reported to bind nucleic acids in vitro by targeting sequences with a central C(N)CAUC motif in RNA through its cold shock domain [37, 38], which is also found in circSERPINE2. We used the catRAPID algorithm to analyze the binding affinity of circSERPINE2 for YBX3 and found that 2 regions of YBX3 are predicted to have high interaction capacity with circSERPINE2: YBX3 residues 101–152 and 247–298 (Fig. 4A and Fig. s2H). As determined by RNA-binding protein immunoprecipitation assay and RNA pulldown assay, circSERPINE2 and YBX3 binding requires the presence of the 101–152 region (Fig. 4B, C). In addition, the catRAPID algorithm demonstrated that the regions of circSERPINE2 containing the C(N)CAUC motif (34–38 bp, 196–200 bp) were predicted to possess high affinity for YBX3. Thus, we constructed a plasmid expressing circSERPINE2 with specific deletion of the sequences through which circSERPINE2 might bind with YBX3 (Δ34-38 and Δ196-200). The overexpression efficiencies of the wild-type (WT) plasmid and deletion (DEL) plasmid were comparable (Fig. 4D). Compared with truncated circSERPINE2 and the vector control, unmodified circSERPINE2 exhibited stronger binding with YBX3 in HEK293T cells in both the RNA pulldown assay and RIP (Fig. 4E and F, Fg. S2I). Besides, co-IP assay demonstrated that overexpression of wild-type circSERPINE2 cemented the interaction between YBX3 and ZO-1, which was not observed for overexpression of truncated circSERPINE2 (fig. S2J-K). Accordingly, circSERPINE2 (Δ34-38 and Δ196-200) lost the ability to retain YBX3 in the cytoplasm (Fig. 4G), and MSCs transfected with the DEL plasmid exhibited a lower percentage of SA-β-gal- and p21-positive cells and a higher EdU-positive cell rate than those transfected with WT circSERPINE2 (Fig. 4H–K). Collectively, these data indicate that circSERPINE2 binds YBX3 via the CCAUC motif and plays an important role in accelerating MSC senescence.

### CircSERPINE2/YBX3 affects the transcription of PCNA, thereby regulating p21 degradation

**Fig. 5:**
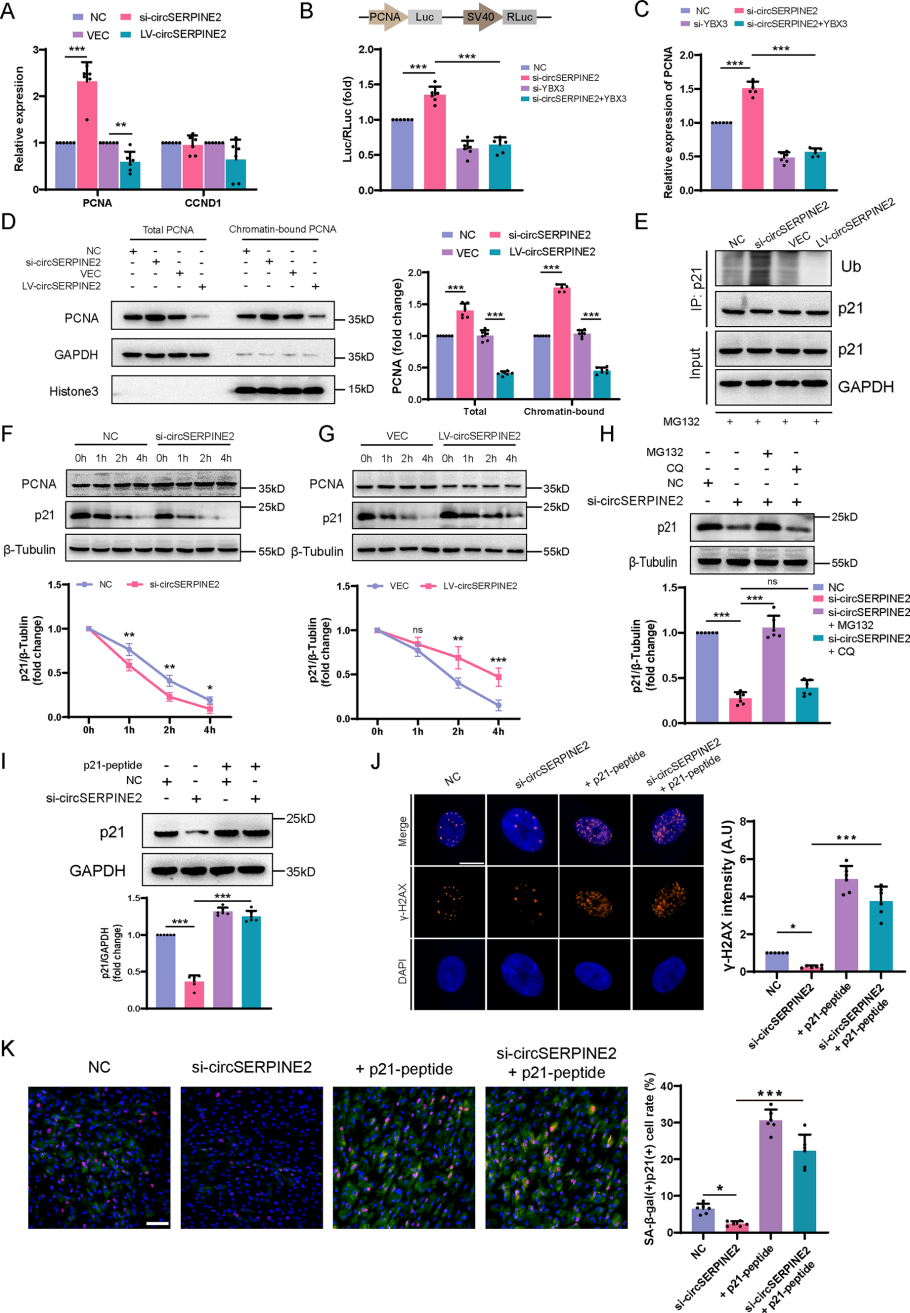
CircSERPINE2/YBX3 impacted PCNA transcription, thereby regulating p21 degradation. **A** The mRNA levels of PCNA and CCND1 in MSCs transduced with si-circSERPINE2 or LV-circSERPINE2 were analyzed by RT–qPCR. The data are presented as the mean ± SD; *n* = 6 biological replicates. **P < 0.01, ***P < 0.001 (two-tailed *t* test). **B** Schematic of the PCNA promoter reporter construct (pZX-luc-RLUC) used to evaluate PCNA transcription. Luc levels were normalized to RLUC levels in each group; the data were then plotted as the difference in the Luc/RLuc ratio between the NC siRNA transfection group and the other groups. The data are presented as the mean ± SD; *n* = 6 biological replicates. ***P < 0.001 (one-way ANOVA). **C** RT–qPCR analysis of MSCs transfected with NC siRNA, si-circSERPINE2, si-YBX3 or si-circSERPINE2 + si-YBX3. The data are presented as the mean ± SD; *n* = 6 biological replicates. ***P < 0.001 (one-way ANOVA). **D** Total and chromatin-bound PCNA protein levels were determined by immunoblotting, with GAPDH and Histone3 as internal references. The data are presented as the mean ± SD; *n* = 6 biological replicates. ***P < 0.001 (one-way ANOVA). **E** MSCs transfected with the indicated siRNAs or with the indicated LV were treated with MG132 (20 μM) for 6 h before harvest. p21 was immunoprecipitated with an anti-p21 antibody, and the immunoprecipitates were probed with an anti-Ub or anti-p21 antibody. *n* = 3 biological replicates. **F** and **G** si-circSERPINE2 (**E**) or LV-circSERPINE2 (**F**) was transfected into MSCs. After transfection of siRNA or LV for 48 h, protein lysates were prepared at the indicated time points after treatment with 100 ng/ml CHX, and the p21 protein levels were evaluated by western blotting. The data are presented as the mean ± SD; *n* = 3 biological replicates. *P < 0.05, **P < 0.01, ***P < 0.001 versus the control group (two-way ANOVA). **H** MSCs were transfected with NC siRNA or si-circSERPINE2 for 48 h, after which MSCs were treated with CQ (10 µM) or MG132 (20 µM) for another 6 h. The data are presented as the mean ± SD; *n* = 6 biological replicates. ***P < 0.01 (one-way ANOVA). **I** Western blot analysis of MSCs transfected with NC siRNA or si-circSERPINE2 for 48 h and then treated with p21-peptide or not for 24 h. The data are presented as the mean ± SD. *n* = 6 biological replicates. ***P < 0.001 (one-way ANOVA). **J** Immunofluorescence analysis of γ-H2AX in MSCs transfected with NC siRNA or si-circSERPINE2 and p21-peptide or not. The γ-H2AX intensity was determined by ImageJ. The data are presented as the mean ± SD; *n* = 6 biological replicates. *P < 0.05, ***P < 0.001 (one-way ANOVA). γ-H2AX (orange) and DAPI (blue). Scale bar = 10 µm. **K** SA-β-gal staining and p21 immunofluorescence staining of MSCs transfected with NC siRNA or si-circSERPINE2 and p21-peptide or not. Scale bar = 50 µm. SA-β-gal- and p21-positive cells were identified with ImageJ. The data are presented as the mean ± SD; *n* = 6 biological replicates. *P < 0.05, ***P < 0.001 (one-way ANOVA)

Transcriptional regulation of PCNA and CCND1 depends on the nuclear localization of the YBX3 protein [39]. RT–qPCR showed that knockdown of circSERPINE2 increased the mRNA expression of PCNA but not CCND1, while overexpression of circSERPINE2 suppressed PCNA transcription without affecting CCND1 transcription (Fig. 5A). YBX3 has been reported to bind the PCNA promoter at nucleotides -560 to + 60 through an inverted CCAAT box [33]. To confirm whether the change in PCNA transcription was driven by circSERPINE2-YBX3, we constructed a heterologous Luc reporter in which the PCNA promoter drives Luc activity wherein RLuc expressed from the same vector driven by the SV40 promoter served as the transfection control. The dual Luc reporter assay showed that silencing circSERPINE2 significantly elevated PCNA-driven Luc expression and that this effect could be reversed by YBX3 knockdown (Fig. 5B). RT–qPCR further verified the change in PCNA mRNA levels (Fig. 5C). In addition, the truncated circSERPINE2 plasmid exerted no effect on PCNA transcription (Fig. S3A and B). Subsequent western blotting analysis demonstrated that both total PCNA protein levels and the level of chromatin-bound PCNA, the functional part of PCNA that promotes DNA damage repair [40], changed after circSERPINE2 transfection (Fig. 5D). In addition, chromatin-bound PCNA has been reported to improve p21 ubiquitin-mediated degradation [40, 41]. Previous works demonstrated that increase of p21 alone is sufficient to induce cellular senescence [42]. The preceding results indicated that the change in circSERPINE2 expression had a very large impact on p21 protein levels. However, neither circSERPINE2 knockdown nor circSERPINE2 overexpression affected p21 mRNA levels (Fig. S3C). Then, we used MG132 to block the ubiquitin‒proteasome-dependent degradation of p21. Indeed, silencing circSERPINE2 led to a significant increase in p21 polyubiquitylation, whereas overexpression of circSERPINE2 reduced the levels of polyubiquitylated p21 (Fig. 5E). In line with the change in p21 ubiquitylation, the degradation rate of p21 was higher after circSERPINE2 was silenced but lower after circSERPINE2 overexpression in MSCs treated with CHX (Fig. 5F and G). Furthermore, MG132 reversed the degradation of p21 after transfection with si-circSERPINE2, while CQ exerted little effect, indicating that circSERPINE2 could impact p21 degradation through the ubiquitin–proteasome pathway (Fig. 5H). To inhibit the degradation of p21 mediated by PCNA, we designed a canonical p21 peptide that competitively binds PCNA against p21 [43] (Fig. S3D). The downregulation of p21 expression mediated by circSERPINE2 knockdown could be abrogated by inhibition of the interaction between PCNA and p21, further verifying the involvement of the circSERPINE2/YBX3/PCNA/p21 axis in MSC senescence (Fig. 5I). Finally, we further confirmed that PCNA mediated the impact of circSERPINE2 on MSC senescence in vitro. We performed rescue experiments and found that inhibition of the PCNA/p21 complex could reverse the antisenescence effect of circSERPINE2 silencing (Fig. 5J and K). Collectively, these data demonstrate that circSERPINE2 hinders YBX3 translocation, inhibits the transcription of PCNA, and interferes with p21 degradation, ultimately accounting for MSC senescence.

### Misregulated splicing events might account for the accumulation of circSERPINE2 during senescence

**Fig. 6:**
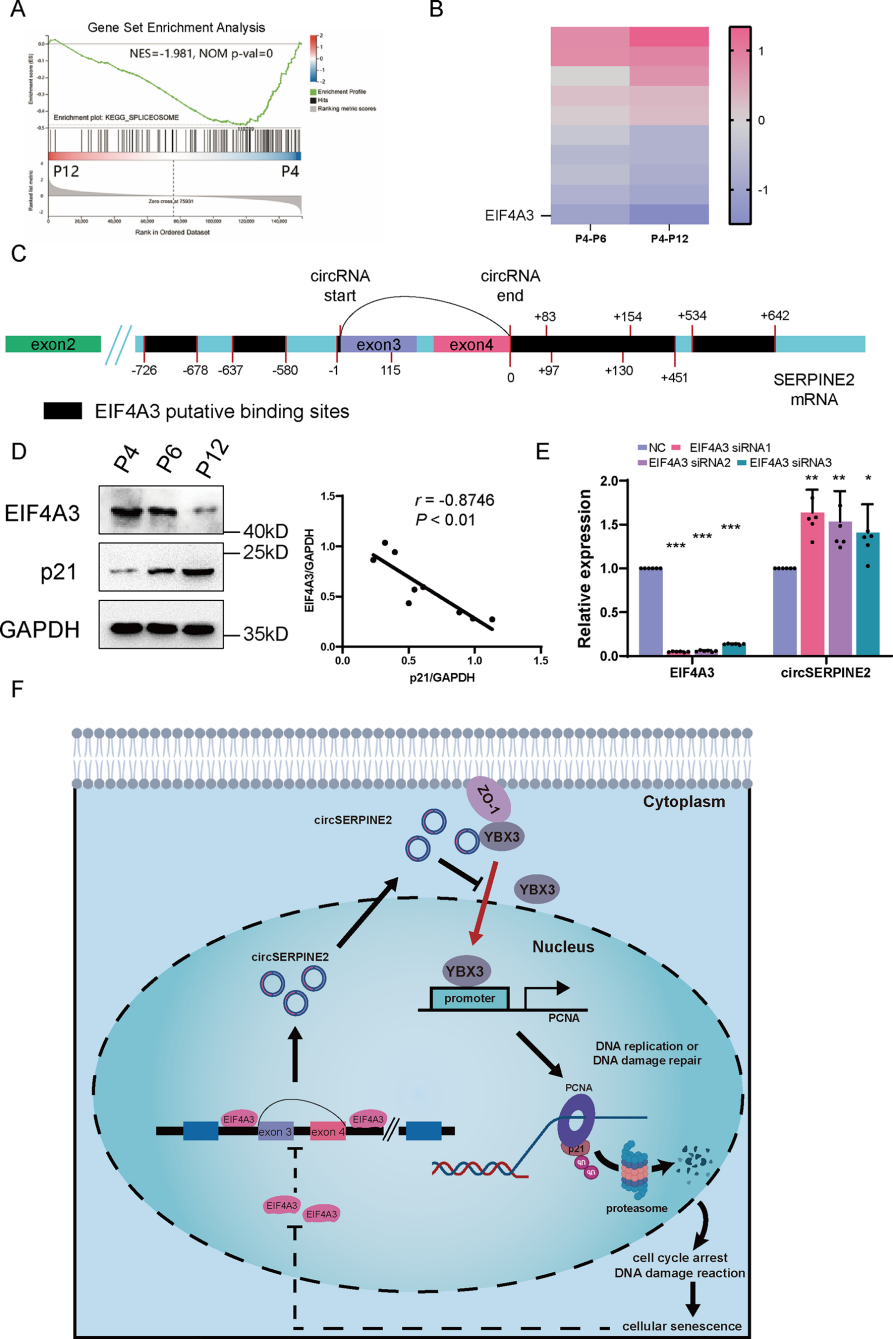
Senescence-related downregulation of EIF4A3 expression promoted circSERPINE2 expression. **A** GSEA enrichment analysis of DE genes between P4 and P12 MSCs in terms of the spliceosome. **B** Heatmap of the expression of top 10 DE genes related to the spliceosome pathway. **C** The binding sites of EIF4A3 in the upstream and downstream regions of the SERPINE2 mRNA transcript were predicted using the circRNA interactome. **D** Western blot analysis revealed that EIF4A3 expression was downregulated in senescent MSCs. The correlation between EIF4A3 and p21 protein levels in P4, P6, and P12 MSCs was analyzed by GraphPad Prism 8.3.0. *n* = 3 biological replicates. **E** RT–qPCR analysis of EIF4A3 in MSCs transfected with NC siRNA or EIF4A3 siRNAs. The data are presented as the mean ± SD; *n* = 6 biological replicates. *P < 0.05, **P < 0.01, ***P < 0.001 (two-tailed *t* test). **F** Putative model showing the role of circSERPINE2 in MSC senescence

Cellular senescence accompanied by misregulated splicing events has been proven to contribute to the circulation of RNAs [44]. Correspondingly, RNA-seq analysis showed that DE mRNAs between P4 MSCs and P12 MSCs were associated with the spliceosome, as demonstrated by Gene Set Enrichment Analysis (GSEA) (Fig. 6A). To determine whether senescence-related misregulated splicing events give rise to circSERPINE2, we selected EIF4A3, the most downregulated splicing factor in late-passage MSCs, for further investigation (Fig. 6B). EIF4A3 is a splicing factor that participates in the splicing of pre-mRNA and has been reported to be related to the circulation of RNAs [45, 46]. We identified seven possible binding sites between EIF4A3 and the flanking regions of circSERPINE2 by using a bioinformatics method (circinteractome.nia.nih.gov), which indicated that EIF4A3 might regulate circSERPINE2 circulation (Fig. 6C and Fig. S4). Western blotting analysis showed that EIF4A3 expression was downregulated in senescent MSCs (Fig. 6D). We then knocked down the expression of EIF4A3 and found an increase in circSERPINE2 expression (Fig. 6E). All of these data confirm that the increase of circSERPINE2 in senescent MSCs might also be attributed to spliceosome dysfunction (Fig. 6F).

### CircSERPINE2-based nucleic acid therapy inhibits the development of aging-related OA in mice by alleviating cellular senescence

**Fig. 7:**
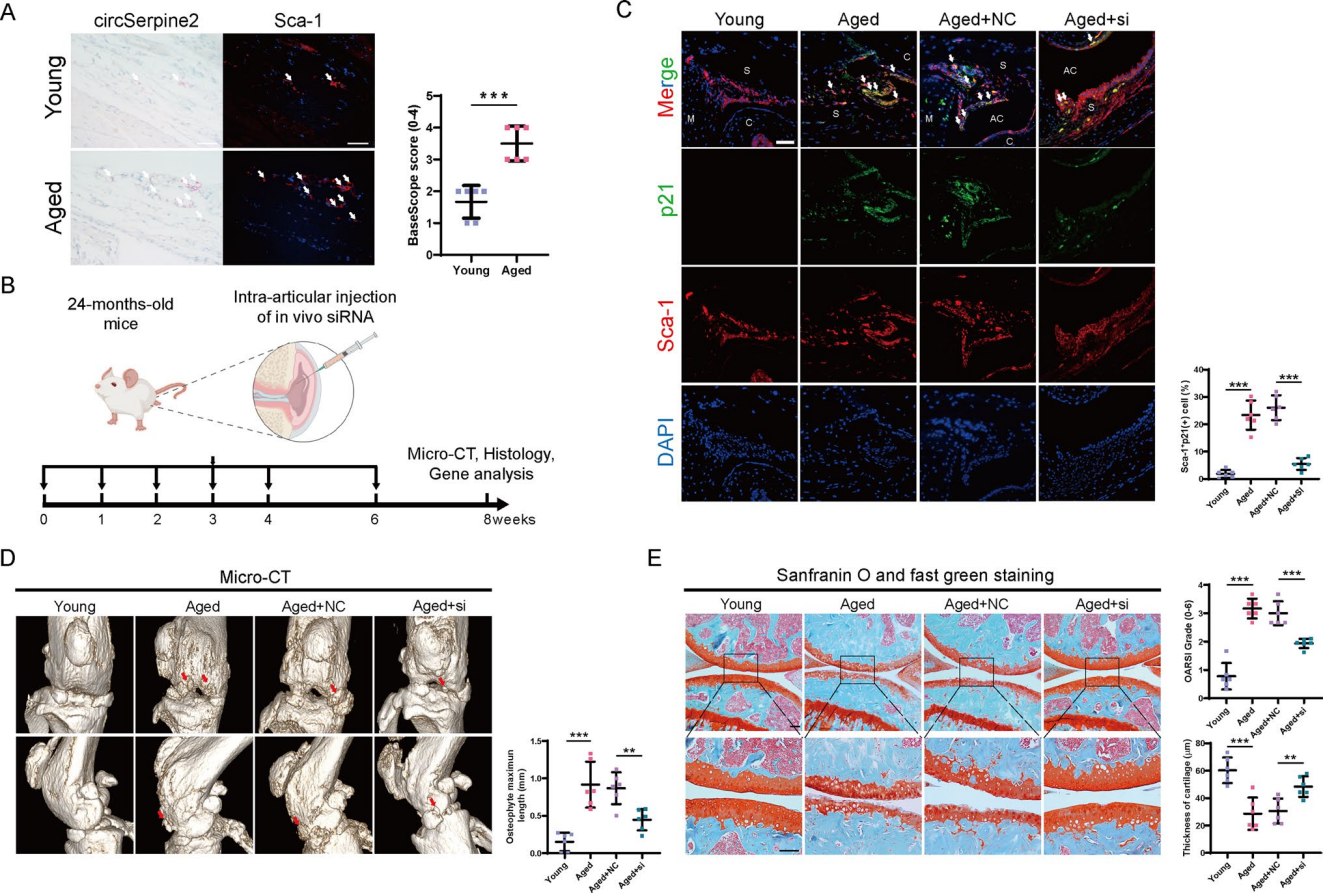
Silencing circSerpine2 in vivo alleviated OA development in mice. **A** Base Scope assay and Sca-1 immunofluorescence staining demonstrated the expression level of circSerpine2 in MSCs in joints of young or aged mice. Left, bright field images showing the Base Scope assay of circSerpine2. Right, immunofluorescence images of Sca-1. The white arrows indicated Sca-1^+^circSerpine2( +) cells. Scale bar = 20 μm. Semi-quantitative analysis of Base Scope assay was done on Sca-1^+^ cells. The data are presented as the mean ± SD; *n* = 6 mice. ***P < 0.001 (two-tailed *t* test). **B** Schematic of the time course of the experiments in (**C**–**E)**. **C** Left, representative images showing immunofluorescence costaining of p21 and Sca-1 from the joints of young mice and aged mice injected with NC siRNA or si-circSerpine2. The white arrows indicated Sca-1^+^p21( +) cells. Right, Statistical analysis of the percentage of Sca-1^+^p21( +) cells in total Sca-1.^+^ cells. Scale bar = 50 μm. *C* cartilage, *M *meniscus; *AC* articular cavity, S synovium. The data are presented as the mean ± SD. *n* = 6 mice. ***P < 0.001 (one-way ANOVA). **D** Micro-CT analysis of osteophyte formation in mouse joints. The red arrows indicated osteophytes. The data are presented as the mean ± SD; *n* = 6 mice. *** *P* < 0.001 (one-way ANOVA). **E** Left, representative images showing Safranin O and Fast Green staining of articular cartilage from the joints of young mice and aged mice injected with NC siRNA or si-circSerpine2. Right panel, OARSI score of articular cartilage and average thickness of arthritic cartilage. Scale bar = 50 μm. The data are presented as the mean ± SD. *n* = 6 mice. *** *P* < 0.001 (one-way ANOVA)

The accumulation of senescent cells in various mammalian tissues has long been known as a contributor to the development and progression of aging-associated diseases, such as OA, the most prevalent musculoskeletal degeneration disorder among elderly individuals [47]. Senescent chondrocytes have been demonstrated to play an important role in the pathophysiology of OA [15]. In addition, recent studies have found that senescent native joint-resident MSCs participate in the development of OA [15, 48, 49]. Therefore, total RNA was extracted from the synovium of OA or non-OA patients, and RT‒qPCR was performed to verify the expression of differentially expressed circRNAs. The results showed that only circSERPINE2 was significantly increased in the synovium of OA patients, which was consistent with our in vitro experiments performed in MSCs at different passages (Fig. S5A). Exons 3 and 4 of SERPINE2 were found to be highly conserved between humans and mice, with a similarity of 84% (Fig. S5B). Then, we verified the backsplicing junction sequence of circSerpine2 by Sanger sequence analysis, which provided powerful evidence of the conservation of circSerpine2 in mice (Fig. S5C-D). The Base Scope assay demonstrated the aging-related accumulation of circSerpine2 in mouse joints and the enrichment of circSerpine2 in synovial Sca-1^+^ MSCs, which was not observed in chondrocytes (Fig. 7A and Fig. S5E). Besides, FISH assay verified the upregulation of circSERPINE2 in OA patients (Fig. S5F). Liu et al. demonstrated that strategies for alleviating MSC senescence could also be gene therapies to attenuate the development of OA by repressing cellular senescence [17, 50, 51]. Therefore, we postulated that knocking down circSERPINE2 could help attenuate the development of aging-related OA. Our preliminary experiments demonstrated that intra-articular injection of 1 nmol of chemically modified siRNA inhibited circSerpine2 expression even after 2 weeks (Fig. S5G). Physiologically aged mice (24 months old) were injected with NC siRNA or circSerpine2 siRNA once a week for five weeks and twice a week for one week. The mice were sacrificed 8 weeks after the first injection (Fig. 7B). Immunofluorescence revealed that the expression of the cellular senescence marker p21 was downregulated in Sca-1^+^ MSCs in the synovium, indicating the beneficial role of knocking down circSerpine2 in alleviating MSC senescence (Fig. 7C). Micro-CT revealed that circSerpine2-based nucleic acid therapy diminished osteophyte formation and bone damage in the joints (Fig. 7D). In addition, silencing circSerpine2 inhibited cartilage degradation and decreased the OARSI grade (Fig. 7E). Besides, immunofluorescence staining of YBX3 and PCNA showed that knocking down circSerpine2 did not affect the expression of YBX3 but significantly increased the level of PCNA, which was consistent with the in vitro experiments (Fig. S5H). Altogether, these findings confirm that si-circSERPINE2 exhibits therapeutic potential for degenerative joint diseases by alleviating joint-resident MSC senescence.

## Discussion

In the present study, we found for the first time that circSERPINE2 is highly expressed in aging MSCs and facilitates the development of MSC senescence. Mechanistically, circSERPINE2 accumulates during MSC senescence and sequesters YBX3 in the cytoplasm, thus blocking its transcriptional regulation of PCNA and ultimately preventing p21 ubiquitin-mediated degradation. Moreover, the most exciting finding of this study is that si-circSerpine2 has promising therapeutic potential in relieving cellular senescence and treating aging-associated tissue degeneration, such as OA, in vivo.

It has long been demonstrated that circRNAs accumulate in aging tissues and nonproliferating cells, such as terminally differentiated neurons, owing to their high stability in vivo [52]. However, few studies have determined whether circRNA levels are high and upregulated in senescent stem cells. Our previous results showed that the expression of the vast majority of DE circRNAs between early-passage MSCs and late-passage MSCs is upregulated in late-passage MSCs, suggesting that circRNAs can also accumulate in senescent MSCs and might play a vital role in regulating MSC senescence [27]. A relationship between circRNAs and cellular senescence has been identified in fibroblasts, cardiomyocytes, endothelial cells, skeletal muscle stem cells and so on [53–56]. However, the role of circRNAs in human MSCs has not been previously investigated. Recent studies on MSC senescence have mainly focused on epigenetic regulation, which includes histone modification and microRNAs [57]. To our knowledge, this is the first evidence that circRNAs are tightly associated with MSC senescence. Our results prove that circSERPINE2 accumulates during the development of MSC senescence and accelerates MSC senescence. However, we speculate that the high stability of circRNAs might not be the only reason for the abundance of circRNAs and upregulation of their expression in senescent cells. In addition to reduced turnover, the high efficiency of circRNA production contributes to their steady-state abundance [28]. Previous work in *Drosophila melanogaster* cells has shown that inhibiting the spliceosome by deleting splicing factors markedly increases the levels of circRNAs [58]. Interestingly, the expression of core spliceosome components and splicing factors has been reported to be dysregulated during organism aging and cellular senescence [59–61]. Whether dysregulation of pre-mRNA processing events during senescence contributes to the growth of circRNAs remains unclear. Our results prove that EIF4A3 expression is downregulated in senescent MSCs and that silencing EIF4A3 upregulates circSERPINE2 expression, suggesting the possible role of dysregulated splicing events in circRNA production. However, whether other splicing factors dysregulated during cellular senescence are related to circSERPINE2 expression requires further investigation.

CircRNAs often interact with RBPs to exert their biological effects. In this study, we investigated the mechanism through which circSERPINE2 accelerates MSC senescence by binding with YBX3. As an important transcription factor involved in cell proliferation, YBX3 is associated with epithelial cell differentiation and hepatic ischemia/reperfusion injury [62, 63]. In previous studies, YBX3 was found to induce epithelial cell proliferation by increasing PCNA and CCND1 transcription [33], suggesting that YBX3 is an essential regulator of cell proliferation capacity. Moreover, our results demonstrate that the nuclear translocation of YBX3 is blocked by reinforced binding with ZO-1 through an interaction with a special motif in circSERPINE2, causing the cytoplasmic retention of YBX3 and reducing the transcription of PCNA. PCNA is a cofactor of DNA polymerase delta and plays an important role in DNA replication and repair in response to DNA damage, acting as a ring-shaped homotrimer that slides along DNA [64]. A recent study revealed that extracellular vesicles can deposit PCNA mRNA to rejuvenate aged bone marrow-derived MSCs and slow aging-related tissue degeneration [65]. Moreover, a clinical study found that a hypomorphic PCNA mutation can lead to neurodegeneration and premature aging, further suggesting a close relationship between PCNA and senescence [66]. Senescent cells are characterized by cell cycle arrest and activation of the DNA damage response. In the present study, we demonstrated that chromatin-bound PCNA rejuvenated senescent MSCs by regulating ubiquitin-dependent degradation of p21, which has long been known as a regulator of cell cycle progression and DNA damage repair [67, 68]. The alleviation of senescence induced by knockdown of circSERPINE2 could be reversed by disrupting the interaction between PCNA and p21, indicating that the antisenescence effect of circSERPINE2 knockout could be at least partially explained by the degradation of p21 mediated by PCNA.

OA is an aging-related disorder characterized by cartilage attrition, synovitis and osteophyte formation. Recently, studies have found that the accumulation of senescent articular chondrocytes and joint-resident MSCs results in a senescent joint microenvironment and OA [49, 69]. Cao proposed that senescent chondrocytes promote MSC senescence and impair their cartilage regeneration capacity [70], while Malaise indicated that senescent MSCs lose their seno-suppressive paracrine effect toward chondrocytes and then contribute to OA development [71]. These studies demonstrate the intricate pro-senescence positive feedback in the pathology of OA and suggest the therapeutic potential of alleviating MSC senescence. Liu and his team verified that strategies aimed at restraining MSC senescence exhibit outstanding potential in treating aging-related cartilage degeneration [17, 50, 51]. In line with these findings, our results demonstrated that there were fewer senescent MSCs and chondrocytes and that there was less cartilage destruction after intra-articular injection of si-circSerpine2. However, in vitro experiments demonstrated that transfection of si-circSerpine2 exerted no impact on mouse chondrocyte senescence, sequestration of YBX3 or the protein level of p21 (Fig. S6A−D). In addition, Base Scope assay demonstrated that circSerpine2 expression was relatively low in chondrocytes and had no difference between young or senescent chondrocytes. Therefore, we supposed that si-circSerpine2 exerts little impact on arthritic chondrocytes because of their low expression of circSerpine2. Previous studies have found that senescent MSCs can accelerate chondrocyte senescence through their paracrine properties [71]. Therefore, the alleviation of chondrocyte senescence might be the result of the antisenescence effect on MSCs instead of the direct impact exerted by si-circSerpine2, which needs further investigation in the future. Interestingly, overexpression of another circRNA of SERPINE2 cyclized by exons 2 to 4 can alleviate OA [72].

Local clearance of senescent cells or alleviation of cellular senescence was shown to have an outstanding therapeutic effect in mice with OA [50, 73]. Currently, preventing cellular senescence through in situ injection of an LV or adeno-associated virus (AAV) expressing rejuvenating factors is regarded as an effective approach for treating age-related or posttraumatic OA in mice [51]. However, few studies have proven the therapeutic effect of nucleic acid-based therapies for OA. siRNA-based therapies have recently emerged as beneficial therapeutic strategies, as demonstrated by the recent approval of various nucleic acid-based therapeutics by the United States Food and Drug Administration (FDA) as well as the European Medicines Agency (EMA) [74]. Moreover, they are more promising for development into drugs through the advancement of bioengineering materials [75]. Our study provides powerful evidence that nucleic acid therapy with a chemically modified siRNA targeting circSerpine2 has a suppressive effect on the accumulation of senescent cells in the articular cavity and the development of OA, which strongly supports the value of siRNA-based therapies targeting circSERPINE2 for OA.

There are still some limitations to this study. First, we cannot conclude whether the observed downregulation of the expression of the linear transcript SERPINE2 was caused by off-target effects during knockdown of circSERPINE2, although we confirmed that SERPINE2 exerts no effect on MSC senescence. Several articles and reviews have claimed that circRNAs can affect parental gene transcription [76–78]. Therefore, we speculate that circSERPINE2 might promote SERPINE2 expression in some ways not related to MSC senescence. Second, we did not explore the role of motif (34–38 bp or 196–200 bp) alone in the interaction between circSERPINE2 and YBX3. However, our results proved that deletion of the UCCAUCA motif (195–201 bp), which includes the CCAUC motif (196–200 bp), exerted no effect on the interaction between circSERPINE2 and YBX3. Therefore, we hypothesized that the interaction between circSERPINE2 and YBX3 might depend on both motifs (34–38 bp and 196–200 bp) instead of one of them. Third, although we did not explore the exact mechanism underlying the associations among circSERPINE2, YBX3 and ZO-1, we speculate that circSERPINE2 enhances the interaction between YBX3 and ZO-1 by acting as a scaffold. In addition, the mechanism by which dysregulation of the splicing factor EIF4A3 mediates circSERPINE2 circulation requires further investigation. At last, previous work hypothesized that MSCs resident in other niches, such as synovium, were migrated from the bone marrow [15]. Though native joint-resident MSCs were the major target of si-circSerpine2 in vivo, while bone marrow-derived MSCs were used for in vitro experiments, we thought that they shared the same molecular mechanism axis regulated by circSERPINE2. The results of Base Scope assay and p21 immunofluorescence confirmed our hypothesis.

Taken together, our findings provide novel insights linking circSERPINE2 with MSC senescence and highlight the therapeutic potential of si-circSERPINE2 for the treatment of aging-related syndromes in vivo.

## Materials and methods

### Isolation and culture of MSCs

MSCs were extracted and cultured as previously described [79, 80]. Briefly, MSCs were isolated and purified from bone marrow using density gradient centrifugation at 12,000 rpm for 30 min and then resuspended in Dulbecco’s modified Eagle’s medium (DMEM, 1000 mg/L glucose, Gibco, Grand Island NY, USA) supplemented with 10% fetal bovine serum (FBS, Hangzhou Sijiqing Biological Engineering Material Company, Limited, Hangzhou, China). Then, the MSCs were seeded in flasks and cultured at 37 °C in 5% CO2. The culture medium was discarded after 48 h to remove nonadherent cells and then replaced every 3 days thereafter. When they reached 80–90% confluence, the MSCs were digested with 0.25% trypsin containing 0.53 mM ethylenediaminetetraacetic acid (EDTA) and reseeded in new flasks; these cells were considered passage 1 cells. The MSCs were expanded and used for subsequent experiments at passages 4 (early passage), passages 6 (middle passage) and passages 12 (late passage).

### Culture of 293 T cells

293 T (human embryonic kidney) cells were cultured in high-glucose DMEM (4500 mg/L glucose, Gibco) containing 10% FBS in an incubator at 37 °C in 5% CO2. When the 293 T cells reached 80–90% confluence, they were digested with 0.25% trypsin containing 0.53 mM EDTA and reseeded in new flasks.

### Human joint synovium specimens

Human non-OA synovium was obtained from patients undergoing total knee replacement surgery, while human non-OA synovium was obtained from patients who had suffered traffic accidents, with no history of arthritic disease. The characteristics of the study subjects are presented in Supplementary Table 1.

### RNA extraction, reverse transcription, and quantitative real-time PCR

Total RNA was extracted from mouse joints or cultured human MSCs using TRIzol (Accurate Biology, Hunan, China). The RNA was reverse-transcribed into cDNA using an Evo M-MLV RT Kit (Accurate Biology) according to the manufacturer’s protocols. Nuclear RNA and cytoplasmic RNA were separated according to the manufacturer’s instructions (Thermo Fisher Scientific NE-PER kit, Thermo Scientific™, MA, USA). Quantitative real-time PCR was performed on a 7500 Real-Time PCR System (Thermo Fisher Scientific) using a SYBR® Green Premix Pro Taq HS qPCR Kit (Accurate Biology). The relative expression levels of each gene were determined by using the 2^−ΔΔCt^ method with GAPDH or Gapdh as the housekeeping gene. Data File S2 shows the forward and reverse primers for each gene or circRNA.

### Protein extraction

Cells were washed three times with PBS and then lysed in RIPA buffer (CWBIO, Jiangsu, China) supplemented with protease inhibitors (CWBIO) and phosphatase inhibitors (CWBIO) for 30 min on ice. Total protein was acquired by centrifuging the lysates at 14,000 rpm for 10 min at 4 °C. The soluble material was collected, and the protein concentration was measured using a Pierce BCA protein assay kit (Thermo Scientific™). Nuclear and cytoplasmic proteins were separated with NE-PER™ Nuclear and Cytoplasmic Extraction Reagents (Thermo Scientific™) according to the manufacturer’s instructions, and the protein concentration was determined as mentioned above.

### Chromatin fractionation

The chromatin-derived fraction was separated as previously reported [81, 82]. First, cells were lysed with buffer A (100 mM NaCl, 300 mM sucrose, 3 mM MgCl2, 10 mM PIPES (pH 6.8), 1 mM EGTA, and 0.2% Triton X-100; containing protease and phosphatase inhibitors) for 30 min on ice. Then, the chromatin-containing pellet was separated from the soluble fraction by centrifuging crude lysates at 14,000 rpm at 4 °C for 10 min. The pellet was digested with RIPA buffer containing phosphatase inhibitor, protease inhibitor and 125 U of benzonase (Yeasen, Shanghai, China) for another 40 min to acquire chromatin-bound proteins. The chromatin-containing supernatants were clarified by centrifugation at 14,000 rpm and 4 °C for 10 min to remove debris, and the protein concentration was quantified by a Pierce BCA protein assay kit (Thermo Scientific™).

### Western blot analysis

Equal amounts of each protein diluted in 5 × sodium dodecyl sulfate (SDS) loading buffer (Beyotime, Shanghai, China) were separated using SDS–polyacrylamide gel electrophoresis (PAGE) and subsequently transferred to polyvinylidene fluoride (PVDF) membranes (Millipore, Massachusetts, USA). After being blocked at room temperature for 1 h, the membranes were incubated with primary antibodies against GAPDH (1:1000, Cell Signaling Technology, Massachusetts, USA, 5174), p21 (1:1000, Abcam, Cambridge, UK, 109,520), p53 (1:1000, Cell Signaling Technology, 48,818), p16 (1:1000, Abcam, 51,243), SERPINE2 (1:1000, Abcam, 134,905), YBX3 (1:2000, Bethyl, Montgomery, USA, A303-070A), β-Tubulin (1:1000, Cell Signaling Technology, 2128), PCNA (1:1000, Abcam, 29), Histone3 (1:1000, Abcam, 176,842), ubiquitin (1:1000, Abcam, 134,953), HNRNPC (1:1000, Abcam, 133,607), YBX1 (1:1000, Proteintech, Rosemont, USA, 20,339–1-AP), and ZO-1 (1:1000, Proteintech, 21,773–1-AP) overnight at 4 °C. The membranes were washed 3 times, incubated with corresponding horseradish peroxidase (HRP)-conjugated secondary antibodies (1:3000, BOSTER, California, USA, BA1050, BA1054) for 1 h at room temperature and then washed 3 times with TBST. Specific immunoreactive bands were detected using Immobilon Western Chemiluminescent HRP Substrate (Millipore). The mean intensity ratio was analyzed by ImageJ 1.4.

### H_2_O_2_-induced senescence

P4 MSCs were seeded in 12-well plates at a density of 0.7 × 10^5^ cells/well. Then, the cells were treated with 600 µM H_2_O_2_ for 2 h to induce senescence. Subsequent experiments were performed on day 7 after induction.

### RNA fluorescence in situ hybridization (FISH)

RNA FISH was performed with the Fluorescent in Situ Hybridization Kit (RiboBio, Guangzhou, China) according to the manufacturer’s instructions. First, MSCs were seeded in 12-well plates, covered with sterile glass, and cultured overnight. Then, the cells were fixed with 4% paraformaldehyde for 10 min and permeabilized with cold 0.5% Triton for another 5 min. After being rinsed 3 times, the MSCs were prehybridized with prehybridization buffer for 30 min at 37 °C. A Cy3-labeled circSERPINE2 probe was constructed by RiboBio (Guangzhou, China). The probe was dissolved in hybridization buffer at a concentration of 20 µM. Then, the slides were hybridized with hybridization buffer overnight. After sequential washes to remove the unconjugated probe, the cells were stained with 4′,6-diamidino-2-phenylindole (DAPI) and observed under an LSM 880 laser scanning confocal microscope. The probe sequence is shown in Supplementary file1.

### RNA stability assays

RNA was treated with RNase R according to the manufacturer’s instructions. Briefly, 2 µg of total RNA was incubated with or without 1 U/µg RNase R (Geneseed, Guangzhou, China) at 37 °C for 0, 5, 10, or 20 min. After inactivation of the enzyme, the treated RNA was reverse transcribed, and equal amounts of cDNA were subjected to agarose gel electrophoresis. For the actinomycin D assay, MSCs were seeded in 12-well plates and treated with actinomycin D (MDbio, Qingdao, China) at a concentration of 20 μg/ml for 0, 8, 12, or 24 h. At each time point, total RNA was extracted and subjected to RT–qPCR as described above.

### Senescence-associated beta galactosidase (SA-β-gal) staining assay

MSCs were seeded in confocal dishes. SA-β-gal staining of MSCs was performed with a Cellular Senescence Detection Kit-SPiDER-βGal (Dojindo, Shanghai, China) according to the manufacturer’s instructions. First, MSCs were treated with bafilomycin A1 for 1 h to block the activity of endogenous β-galactosidase. Then, cells were incubated with SPIDER-*β*Gal working solution for 30 min in 37 °C, 5% CO_2_. After that, cells were co-stained with anti-p21 antibody (1:200, Proteintech, 27,296–1-AP). Thereafter, the dishes were viewed under a fluorescence microscope, and images were obtained using a Leica DMi8. SA-β-gal-positive and p21-positive cells were analyzed with ImageJ software 1.4.

### 5-Ethynyl-2′-deoxyuridine (EdU) incorporation assay

MSCs were seeded in 24-well plates at a density of 0.4 × 10^5^ cells/well. MSCs were transfected with siRNA or lentivirus (LV), and then the proliferation of MSCs was assessed on different days after transfection by fluorescence microscopy using a BeyoClick™ EdU Cell Proliferation Kit (Beyotime) according to the manufacturer’s protocol. EdU-positive cells were identified and counted by ImageJ software 1.4.

### Clonal expansion assay

MSCs were seeded in 12-well plates at a density of 4000 cells per well. MSCs were transfected with siRNA or LV and cultured for another 14 days. Then, the cells were fixed with 4% PFA for 10 min and stained with 0.2% crystal violet (Beyotime) for 1 h at room temperature. Clonal expansion ability was measured by ImageJ 1.4.

### Cell cycle analysis

Cells were seeded in 6-well plates at a density of 1.4 × 10^5^ cells/well. MSCs were transfected with siRNA for 48 h or LV for 7 d. MSCs were collected and fixed with 75% ethanol at -20 °C for 1 h. Then, cells were permeabilized by using Intracellular Staining Perm Wash Buffer (BioLegend, San Diego, USA) overnight at 4 °C. The cells were then washed twice with PBS and stained with PI (BD Pharmingen™, New Jersey, USA, 550,825) at room temperature for 15 min. Cell cycle distribution was measured with a Becton–Dickinson FACSCelesta System (Becton–Dickinson, San Jose, USA).

### siRNA transfection

Three circSERPINE2-, YBX3-, SERPINE2-, and EIF4A3-specific siRNAs and one siRNA of hsa_circ_0058476 and hsa_circ_0019233 were designed and synthesized by IGE Biotechnology (Guangzhou, China). P6 MSCs were transfected with the siRNAs for 48 h using Lipofectamine RNAi MAX (Thermo Scientific™) according to the manufacturer’s protocol. The siRNAs with the best knockdown efficiency were used in subsequent experiments. The sequences of the siRNAs are shown in Supplementary file1.

### Plasmid construction and transfection

Expression plasmid constructs, including pLC5-ciR (Homo sapiens), full-length pLC5-ciR-circSERPINE2, and pLC5-ciR-circSERPINE2 with deleted domains (34–38 bp, 196–200 bp deletion), were constructed by Geneseed (Guangzhou, China). Full-length pcDNA3.1( +)-SERPINE2, pcDNA3.1( +), Flag-pcDNA3.4( +)-YBX3, Flag-pcDNA3.4( +)-YBX3-DEL1(Δ101-152), Flag-pcDNA3.4( +)-YBX3-DEL1(Δ247-298), Flag-pcDNA3.4( +), HA-pcDNA3.4( +)-ZO-1 and HA-pcDNA3.4( +)-SH3 were constructed by IGE Biotechnology (Guangzhou, China). Transfection was performed using a Lipofectamine 3000 Transfection Kit (Invitrogen, MA, USA) according to the manufacturer’s instructions with minor modifications. Briefly, P6 MSCs were seeded in 12-well plates at a density of 0.8 × 10^5^ cells/well. A total of 0.5 µg plasmid supplemented with 1 µg Lipofectamine 3000 (Thermo Scientific™) and 1 µg p3000 (Thermo Scientific™) mixed in Opti-MEM (Gibco) was added to each well according to the manufacturer’s instructions. 293 T cells were seeded in 12-well plates at a density of 1.5 × 10^5^ cells/well, and 1 µg plasmid supplemented with 2 µg Lipofectamine 3000 and 2 µg p3000 mixed in Opti-MEM was added to each well. After transfection for 8 h, the culture medium was replaced.

### LV construction and infection

A circSERPINE2-overexpression LV and vector control were constructed by and purchased from Obio Technology Corp., Ltd. (Shanghai, China). Briefly, P6 MSCs were infected with LV and 5 µg/ml polybrene at a multiplicity of infection (MOI) of 30 (Obio Technology). Subsequent experiments were performed on day 7 after transfection.

### RNA pulldown and mass spectrometry

Biotin-labeled oligonucleotide probes targeting the junction site of circSERPINE2 and antisense probes were synthesized by RiboBio Co., Ltd. (Guangzhou, China). An RNA pulldown assay was performed with the PureBindingTM RNA–Protein pulldown Kit (Geneseed) according to the manufacturer’s protocol. Briefly, streptavidin-coated magnetic beads were incubated with the biotinylated probes at 4 °C for 1 h. After that, magnetic beads were separated using a magnet and incubated with the eluate from 1.0 × 10^7^ MSCs lysed in lysis buffer for another 2 h at 4 °C. The probe-RNA–protein complex was pulled down and separated using a magnet. The retrieved proteins in the complex were separated from the magnetic beads by boiling for 10 min and then analyzed by western blotting or mass spectrometry (The Medical Research Center of Sun Yat-Sen Memorial Hospital, Sun Yat-Sen University, Guangzhou, China). The probe sequences are shown in Supplementary file1.

### RNA immunoprecipitation assay (RIP)

RIP was performed using an RNA Immunoprecipitation Kit (Geneseed) with antibodies specific for YBX3 (1:100, Bethyl, A303-070A), YBX1 (1:100, Proteintech, 20,339–1-AP), ZO-1 (Proteintech, 21,773–1-AP) or IgG (Cell Signaling Technology, 2729) according to the manufacturer’s instructions. Coprecipitated RNAs and proteins were separated by centrifugation through different columns, and the amount of circSERPINE2 in the eluate was determined by RT–qPCR.

### Immunoprecipitation (IP) and coimmunoprecipitation (Co-IP)

IP and Co-IP were performed using the Dynabeads™ Protein G Immunoprecipitation Kit (Invitrogen) according to the kit protocol. Briefly, protein extracted from MSCs was incubated with an anti-p21 antibody (1:100, Abcam, 109,520), IgG control (1:100, Cell Signaling Technology, 2729) or anti-YBX3 antibody (1:100, Bethyl, A303-070A) at 4 °C for 6 h. Then, protein G agarose beads were added. The samples were incubated overnight, and the immunoprecipitates were collected for western blotting.

### Immunofluorescence staining

MSCs were seeded in confocal dishes. When the cells reached the appropriate confluence, the culture medium was discarded, and the cells were rinsed 3 times with PBS. Then, the cells were fixed in 4% paraformaldehyde for 10 min. After washing, 0.5% Triton X-100 was added for another 10 min at room temperature to permeabilize the cells, and 5% normal goat serum was used to block the cells for half an hour. Primary antibodies against YBX3 (1:100, Bethyl, A303-070A), YBX1 (1:50, Proteintech, 20,339–1-AP), ZO-1 (1:100, Proteintech, 66,452–1-Ig), PCNA (1:100, Abcam, ab29) and γ-H2AX (1:100, Cell Signaling Technology, 9718) were added, and the cells were incubated overnight at 4 °C. After washing the cells 3 times with PBS, they were incubated with a goat anti-rabbit IgG (H + L) cross-adsorbed secondary antibody conjubated with Alexa Fluor™ 488 (1:500, Invitrogen, A-11008) or a goat anti-rabbit IgG (H + L) cross-adsorbed secondary antibody conjugated with Alexa Fluor™ 555 (1:500, Invitrogen, A-21428) for 1 h at room temperature. DAPI (Beyotime) and phalloidin (1:50, Abcam, 235,138) were used to sequentially counterstain the nuclei and the cytoskeleton. Thereafter, the dishes were viewed under a fluorescence microscope or a laser scanning confocal microscope. Images were obtained using a Leica DMi8 or LSM 880 microscope with Airyscan (Carl Zeiss).

### Dual luciferase (Luc) reporter assay

A dual PCNA promoter reporter construct, in which the PCNA promoter drives the expression of Luc and the SV40 promoter drives the expression of Renilla luciferase (RLuc), was obtained from IGE Biotechnology (Guangzhou, China). A total of 0.8 × 10^5^ 293 T cells were preseeded in a 24-well plate and transfected with NC siRNA, NC siRNA + si-circSERPINE2, NC siRNA + si-YBX3 or si-circSERPINE2 + YBX3 for 24 h. Then, the cells were transfected with reporter plasmids using Lipofectamine 3000 and p3000. The cells were harvested 24 h later in lysis buffer (Vazyme, Jiangsu, China), and Luc and GLuc activity was measured by a Dual Luciferase Reporter Assay Kit (Vazyme) following the manufacturer’s instructions.

### p21 degradation assay

MSCs were seeded in 12-well plates at a density of 0.7 × 10^5^ cells/well and transfected with si-circSERPINE2, NC siRNA, LV-circSERPINE2 or vector LV. Protein lysates were harvested at the indicated time points after the addition of cycloheximide (CHX, 100 ng/ml, MedChemExpress, New Jersey, USA). Equal amounts of protein were separated by SDS–PAGE. The levels of p21 were determined by western blot analysis at the indicated time points.

### Inhibition of the autophagy–lysosome and ubiquitin–proteasome pathways

MG132 (MedChemExpress) is a potent proteasome inhibitor used to block ubiquitin–proteasome pathways, while chloroquine (CQ, MedChemExpress) is an autophagy inhibitor used to block autophagy–lysosome pathways. Briefly, MSCs were seeded in 12-well plates at a density of 0.7 × 10^5^ cells/well and transfected with si-circSERPINE2 or NC siRNA. After 48 h, the cells were treated with CQ (10 µM) or MG132 (20 µM) for another 6 h to block the autophagy–lysosome or ubiquitin–proteasome pathway. Then, protein lysates were prepared and subjected to immunoblotting.

### p21-peptide synthesis

The peptide mimic was synthesized by IGE Biotechnology (Guangzhou, China). Briefly, a transmembrane peptide was added to the N-terminus of the peptide, along with BSA modification, to enable it to pass through the cell membrane. The amino acid sequence was as follows: GRKRRQTSMTDFYHSKRRLIFS. MSCs were treated with 2 µM p21-peptide for 24 h after transfection with NC siRNA or si-circSERPINE2 for 48 h.

### Microcomputed tomography (Micro-CT) scanning and osteophyte maximum length measurement

Micro-CT was performed to analyze the structures of the knee joints. The obtained tissues were fixed with 4% polyoxymethylene and then scanned using a Siemens Inveon CT scanner at a resolution of 19 μm. The image data were analyzed using RadiAnt DICOM Viewer software.

### Intra-articular injection of siRNA in aged mice

C57BL/6 male mice aged 8–12 weeks or 24 months were used for experiments. Si-circSerpine2 and NC siRNA for in vivo use were synthesized and chemically modified by RiboBio (Guangzhou, China) to transfect tissues [83]. A total of 1 nmol siRNA dissolved in 10 µl of PBS was intraarticularly injected into aged mice (24 months old) once a week for five weeks and then twice a week for one week until the animals were sacrificed at 8 weeks after the first injection. Young mice (8–12 weeks old) were used as positive controls. The dissected joints were processed for micro-CT scanning and histopathological analysis.

### Tissue immunohistochemistry and immunofluorescence

Knee joint sections from mice were fixed in 4% paraformaldehyde at 37 °C under constant agitation for 3 days and decalcified in 10% EDTA (pH 7.4) at 37 °C for 2 weeks, with the EDTA being replaced every two days. Then, the specimens were embedded in paraffin and cut into 5 µm sections. The slides were stained with Safranin-O Fast-Green (Solarbio, Beijing, China) according to the manufacturer’s protocol. The severity of mouse OA was assessed by three observers blinded to the experimental groups and scored according to the OA Research Society International (OARSI) grading system (grade 0–6) [84]. OARSI scores are presented as the maximum average score for each mouse. Representative Safranin O staining images of the most advanced lesions in each section were selected. Immunofluorescence staining of mouse keen joint sections were performed with a p21 antibody (1:100, Abcam, 188,224), Sca-1 antibody (1:200, Abcam, 51,317) and c-Kit antibody (1:100, Abcam, 256,345).

### Base scope in situ hybridization assay

The knee joint sections from C57BL/6 male mice aged 8–12 weeks or 24 months were fixed with 10% neutral formalin and decalcified by Preserve Rapid Decalcification Solution (Pursuit Bio, Beijing, China). Then, the specimens were embedded, and slices were prepared. The circSerpine2 probe for Base Scope detection was provided by Advanced Cell Diagnostics (ACD). A Base Scope Detection Kit from ACD was used for Base Scope detection. The detection of circSerpine2 in the joints was performed in accordance with the instructions under a light microscope. Semi-quantitative scoring of each specimen was performed according to the Base Scope instruction.

### Statistical analysis

Data were compared between two groups using a 2-tailed Student’s t test, and data were compared among three or more groups by one-way ANOVA or two-way ANOVA followed by Bonferroni’s post hoc test using GraphPad Prism 8.3.0. Quantitative data are presented as the mean ± standard deviation. The *n* values represent the numbers of individuals in each experiment. Statistical significance is indicated in the figures as * (*P* < 0.05), ** (*P* < 0.01) or *** (*P* < 0.001), and *P* values greater than 0.05 were considered statistically nonsignificant (ns).

### Supplementary Information

Below is the link to the electronic supplementary material.

Supplementary file1 (XLSX 14 KB)

Supplementary file2 (XLSX 183 KB)

Supplementary file3 (DOCX 3542 KB)

## Data Availability

The RNA-seq data used in this study are available at GEO Accession Number GSE178514.
