# Supplementary material for: Silencing circSERPINE2 restrains mesenchymal stem cell senescence via the YBX3/PCNA/p21 axis
Source: Cell Mol Life Sci. 2023 Oct 13;80(11):325. doi: 10.1007/s00018-023-04975-6 (PMC10575817; doi:10.1007/s00018-023-04975-6)

**SUPPLEMENTARY FIGURES AND FIGURE LEGENDS**


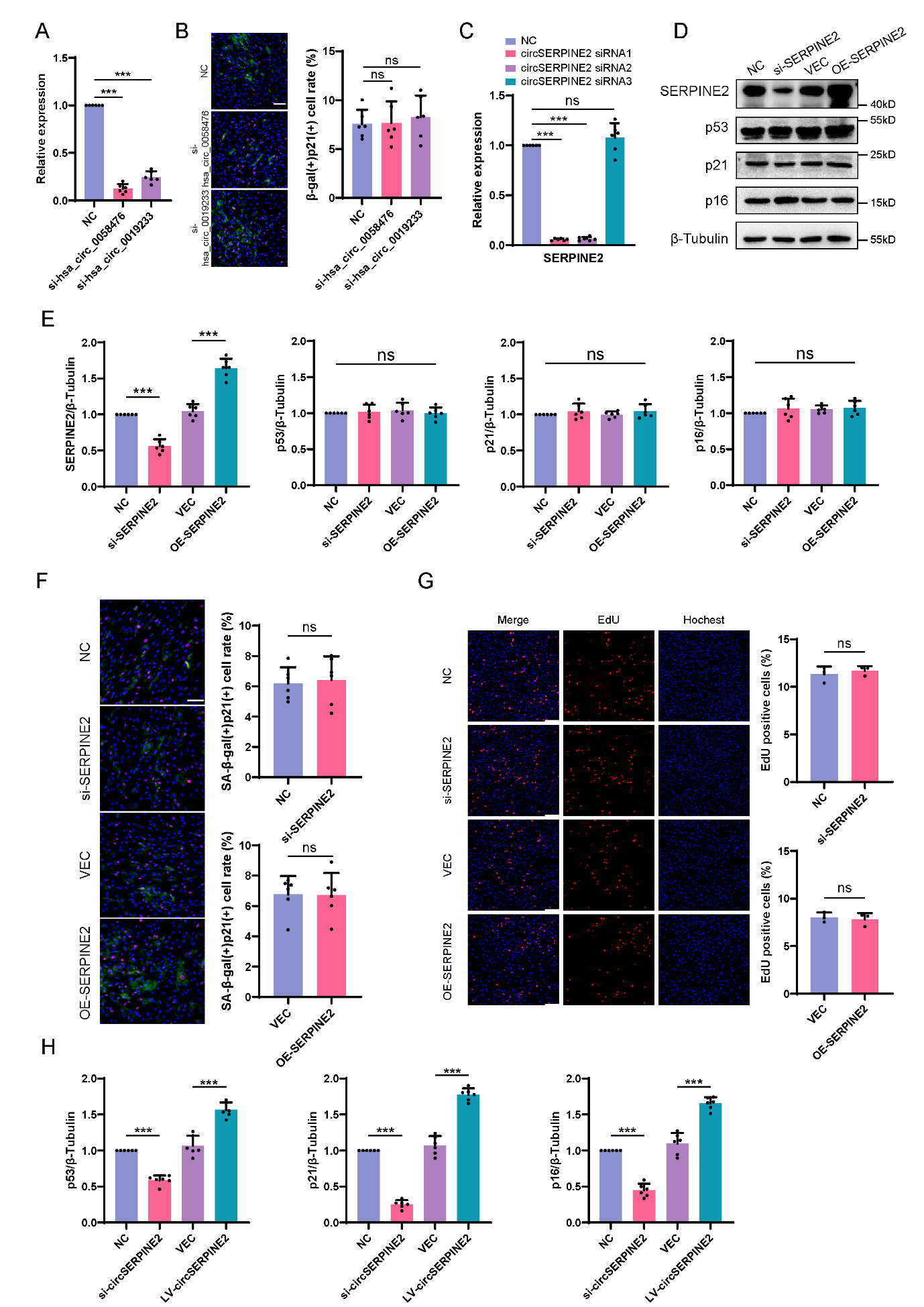


**Supplementary Figure 1. SERPINE2 did not regulate MSC senescence. A**, RT‒qPCR analysis of MSCs transfected with siRNA targeting the junction site of hsa_circ_0058476 or hsa_circ_0019233. RNA levels were normalized to the levels of GAPDH mRNA. The data are presented as the mean ± SD, *n* = 6 biological replicates. ****P* < 0.001 (two-tailed *t* test). **B**, β-Gal staining and p21 immunofluorescence staining of MSCs transfected with NC siRNA, si- hsa_circ_0058476 or si- hsa_circ_0019233. Scale bar = 50 µm. The SA-β-gal- and p21-positive cell rates were determined by ImageJ. The data are presented as the mean ± SD, *n* = 6 biological replicates (one way ANOVA). **C**, RT‒qPCR analysis of MSCs transfected with three siRNAs targeting the junction site of circSERPINE2. RNA levels were normalized to the levels of GAPDH mRNA. The data are presented as the mean ± SD, *n* = 6 biological replicates. ****P* < 0.001 (two-tailed *t* test). **D**, Western blotting of SERPINE2, p53, p21 and p16 proteins in MSCs with silencing of SERPINE2 or overexpression of SERPINE2. β-Tubulin was used as the loading control. *n* = 6 biological replicates. **E**, The relative intensity ratios of the SERPINE2, p53, p21, and p16 proteins were determined by ImageJ with β-Tubulin as a negative control. The data are presented as the mean ± SD, *n* = 6 biological replicates. ****P* < 0.001 (two-tailed *t* test). **F**, β-Gal staining and p21 immunofluorescence staining of MSCs transfected with NC siRNA or si-SERPINE2 for 48 h or transfected with pcDNA3.1(+) or pcDNA3.1(+)-SERPINE2. Scale bar = 50 µm. The SA-β-gal- and p21-positive cell rates were determined by ImageJ. The data are presented as the mean ± SD, *n* = 6 biological replicates (two-tailed *t* test). **G**, EdU staining of MSCs with silencing of SERPINE2 for 48 h or overexpression of SERPINE2. Scale bar = 100 µm. The EdU-positive cell rates were determined by ImageJ. The data are presented as the mean ± SD, *n* = 6 biological replicates. ns indicates *P*>0.05 (two-tailed *t* test). **H**, The relative intensity ratios of p53, p21, and p16 proteins were determined by ImageJ with β-Tubulin as a negative control. The data are presented as the mean ± SD, *n* = 6 biological replicates. ****P* < 0.001 (two-tailed *t* test).


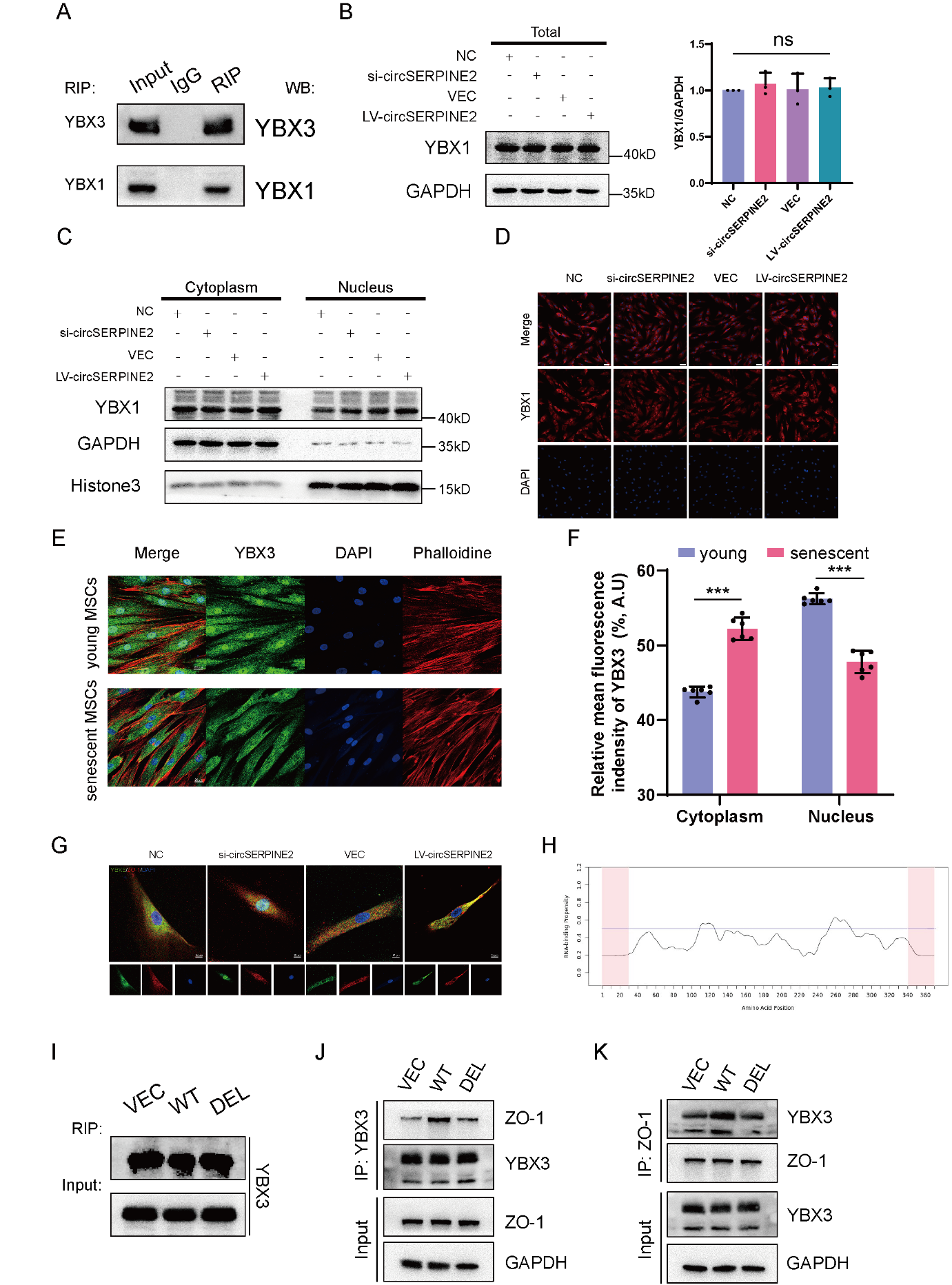


**Supplementary Figure 2. CircSERPINE2 exerted no impact on YBX1 abundance or subcellular location. A,** Western blot assay of proteins from the RIP assay. **B**, Western blot analysis of total YBX1 proteins in MSCs transfected with NC siRNA, si-circSERPINE2, VEC, and LV-circSERPINE2. GAPDH was used as a negative control. *n* = 3 biological replicates. **C**, YBX1 protein levels in cytoplasmic protein and nucleic protein extracts from MSCs transfected with siRNA or lentivirus. GAPDH was the cytoplasmic control, while Histone3 was used as the nucleic control. The data are presented as the mean ± SD, *n* = 3 biological replicates (two-tailed *t* test). **D**, Immunofluorescence of YXB1 (red) and DAPI (blue) in MSCs with knockdown or overexpression of circSERPINE2. **E**, Immunofluorescence of YXB3 (green), DAPI (blue) and phalloidin (red) in young or senescent MSCs. *n* = 6 biological replicates. Scale bar = 20 µm. **F**, Relative immunofluorescence intensity of YBX3 in the cytoplasm or the nucleus of young or senescent MSCs. *n* = 6 biological replicates. ****P* < 0.001 (two-tailed *t* test). **G**, Immunofluorescence colocalization of YBX3 (green) and ZO-1 (red) in MSCs with silencing or overexpression of circSERPINE2. *n* = 6 biological replicates. Scale bar = 10 µm. **H**, The RNA binding propensity between YBX3 and circSERPINE2 was predicted by catRAPID. **I,** Proteins purified from RIP of 293T cells transfected with VEC, WT, or DEL plasmid were determined by western blot analysis. *n* = 3 biological replicates. **J,** Cell lysates were immunoprecipitated with an antibody against YBX3 and analyzed by immunoblotting with an anti-ZO1 antibody or anti-YBX3 antibody. *n* = 3 biological replicates. **K**, Cell lysates were immunoprecipitated with an antibody against ZO-1 and analyzed by immunoblotting with an anti-YBX3 antibody or anti-ZO1 antibody. *n* = 3 biological replicates.


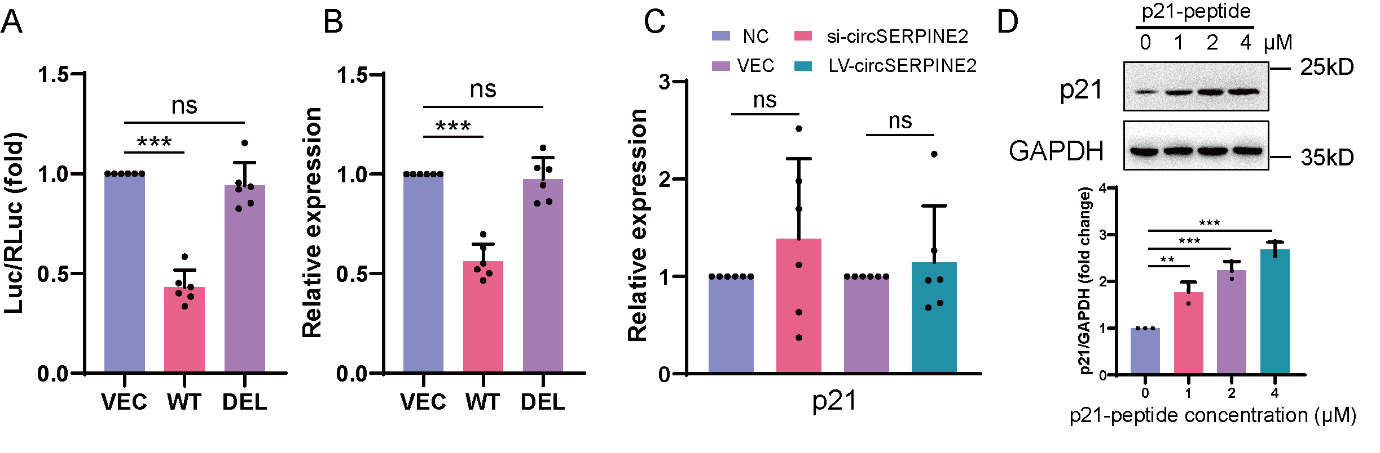


**Supplementary Figure 3. PCNA regulated p21 degradation. A**, Luciferase levels of 293T cells transfected with VEC or WT plasmid or DEL plasmid were determined and normalized to RLUC levels; the data were then plotted as the difference in the Luc/RLuc ratio between the VEC transfection group and the other groups. The data are presented as the mean ± SD; *n* = 6 biological replicates. ****P* < 0.001 (one-way ANOVA). **B**, RT–qPCR analysis of MSCs transfected with VEC or WT plasmid or DEL plasmid. The data are presented as the mean ± SD; *n* = 6 biological replicates. ****P* < 0.001 (one-way ANOVA). **C**, p21 mRNA levels were analyzed by RT‒qPCR in MSCs with circSERPINE2 knockdown or overexpression. The data are presented as the mean ± SD, *n* = 6 biological replicates (one-way ANOVA). **D**, Western blotting analysis of MSCs treated with different concentrations of the p21 peptide. The data are presented as the mean ± SD; *n* = 3 biological replicates. ***P* < 0.01, ****P* < 0.001 (one-way ANOVA).


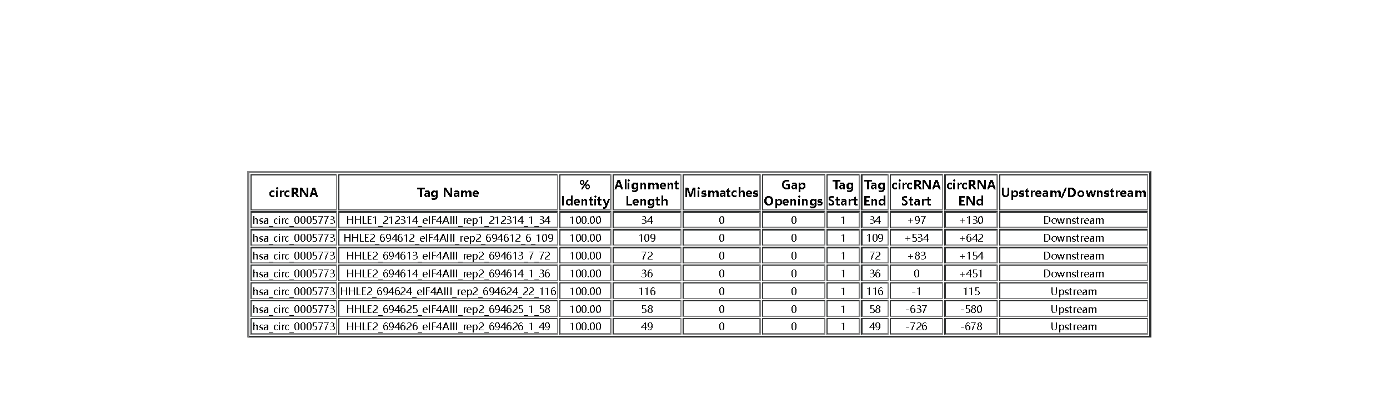


**Supplementary Figure 4. EIF4A3 is predicted to bind flanking regions of circSERPINE2.** The possible binding sites of EIF4A3 in the flanking regions of circSERPINE2 was predicted by CircInteractome.


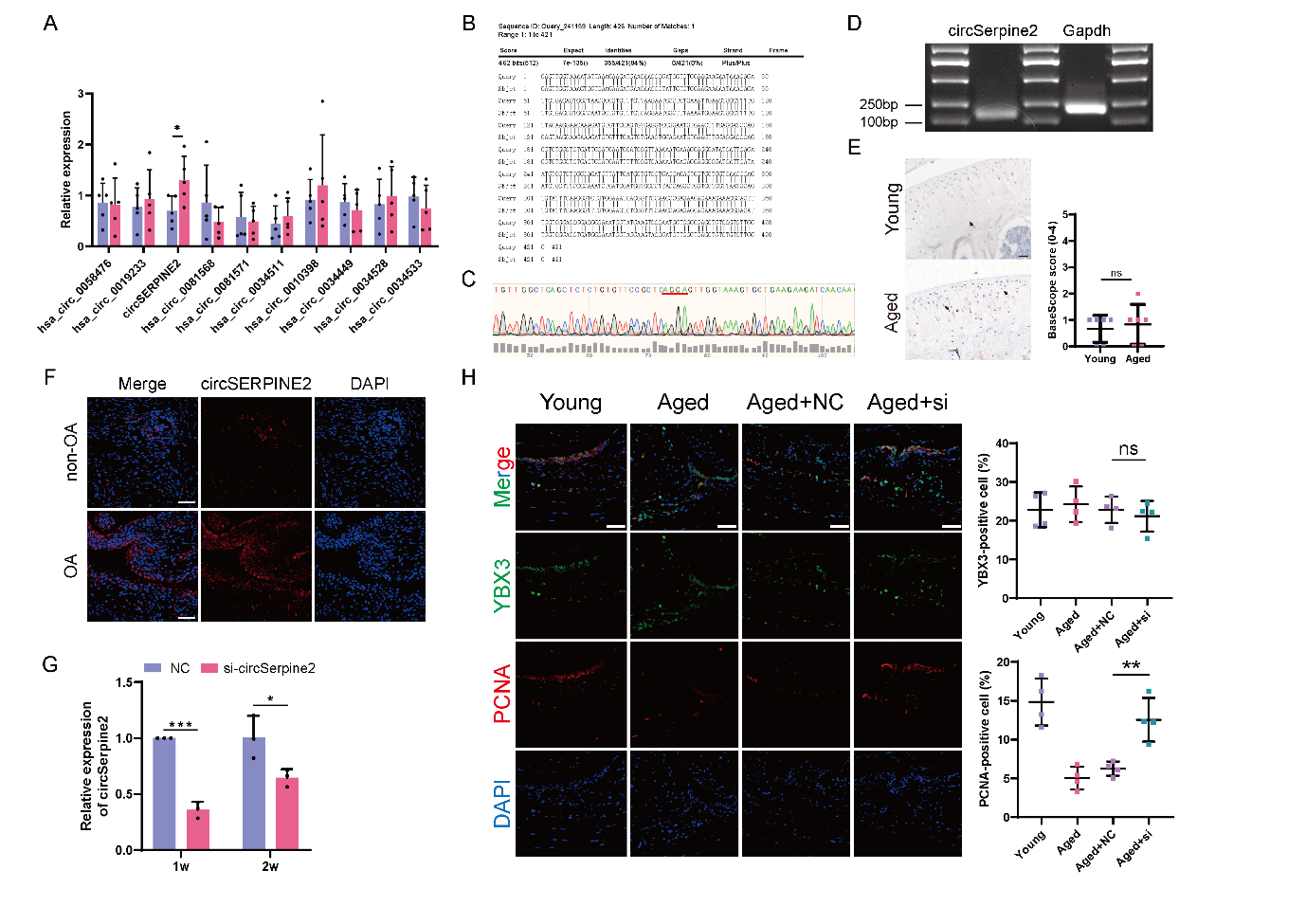


**Supplementary Figure 5. CircSerpine2 is conserved between humans and mice. A**, RT‒qPCR assay showed the expression level of the ten differentially expressed circRNAs in joint synovium from OA or non-OA patients. n = 5 patients in each group. **P* < 0.05 (two-tailed t test). **B**, Similarity of exon 3 and exon 4 of SERPINE2 between humans and mice. **C**, The back-splicing site of circSerpine2 detected by Sanger sequence analysis. **D**, RT‒PCR of products amplified by a specific circSerpine2 divergent primer. **E**, BaseScope assay demonstrated the expression level of circSerpine2 in chondrocytes in joints of young or aged mice. The black arrows indicated circSerpine2(+) chondrocytes. Scale bar = 20 μm. Semi-quantitative analysis of Base Scope assay was done on chondrocytes. The data are presented as the mean ± SD; *n* = 6 mice (two-tailed *t* test). F, FISH assays with probes targeting circSERPINE2 were performed on synovium from non-OA or OA patients. *n* = 5 patients in each group. Scale bar = 50 μm. **G**, RT‒qPCR showed the knockdown efficiency of in vivo si-circSerpine2 in mouse joint tissues after transfection for 1 or 2 weeks. The data are presented as the mean ± SD, *n* = 3 biological replicates. **P* < 0.05, ****P* < 0.001 (two-tailed *t* test). H, Left, representative images showing immunofluorescence costaining of YBX3 and PCNA from the joints of young mice and aged mice injected with NC siRNA or si-circSerpine2. Right, Statistical analysis of the percentage of YBX3-positive or PCNA-positive cells. Scale bar = 50 μm. The data are presented as the mean ± SD. *n* = 4 mice. ***P* < 0.01 (two-tailed *t* test).


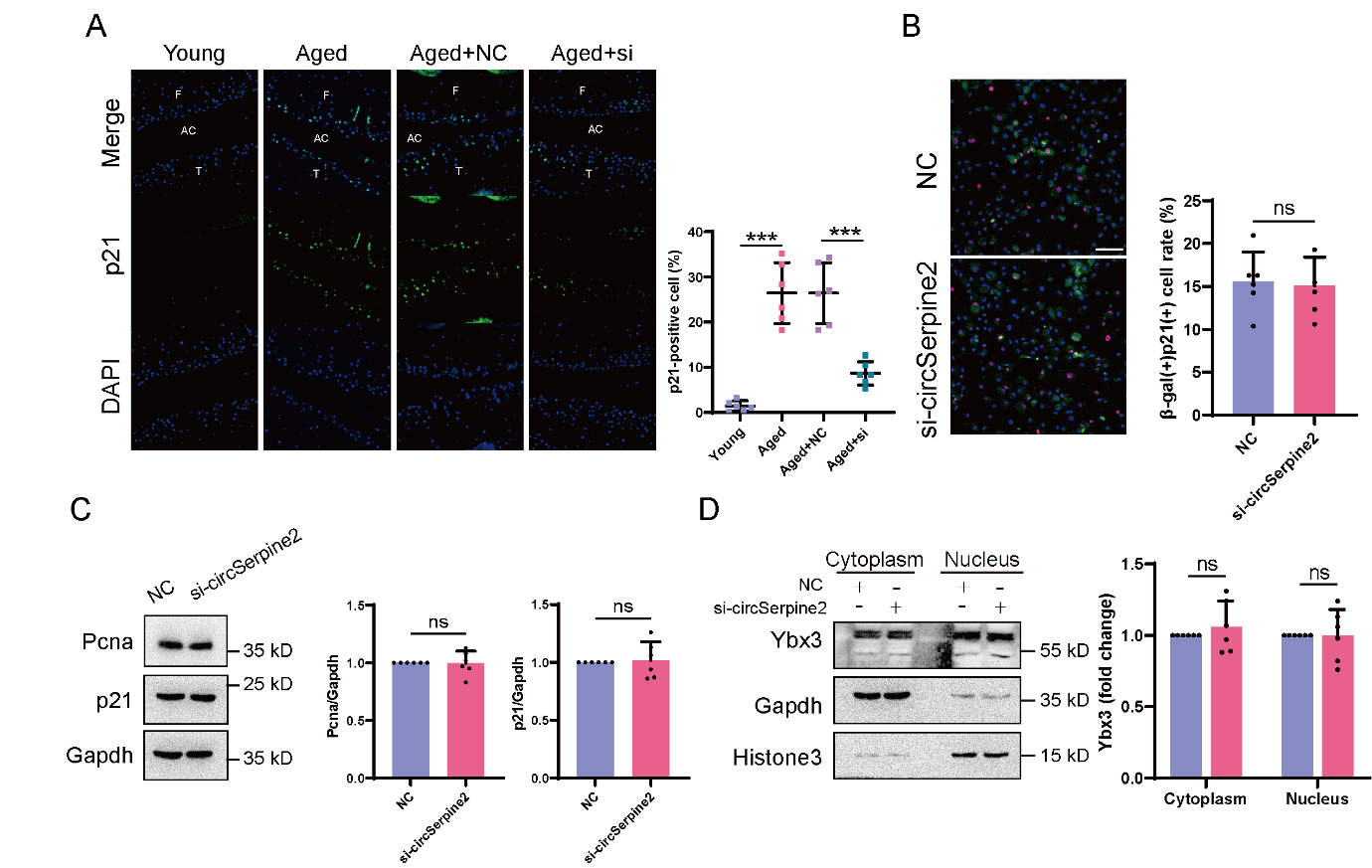


**Supplementary Figure 6. Si-circSerpine2 did not regulate chondrocyte senescence by YBX3/PCNA/p21 axis. A**, Left, Representative images showing immunofluorescence costaining of p21 from the joints of young mice and aged mice injected with NC siRNA or si-circSerpine2. Right, Statistical analysis of p21(+) chondrocytes. The data are presented as the mean ± SD; *n* = 6 mice. *** *P* < 0.001 (one-way ANOVA). F, femur; T, tibia; AC, articular cavity. **B**, β-Gal staining and p21 immunofluorescence staining of chondrocytes transfected with NC siRNA or si-circSerpine2. Scale bar = 50 µm. The SA-β-gal- and p21-positive cell rates were determined by ImageJ. The data are presented as the mean ± SD; *n* = 6 biological replicates.**C**, Western blot analysis of p21 and PCNA in chondrocytes transfected with NC siRNA or si-circSerpine2. *n* = 6 biological replicates. **D**, YBX3 protein levels in cytoplasmic protein and nucleic protein extracts from chondrocytes transfected with siRNA. GAPDH was used as a cytoplasmic control, while Histone3 was used as a nucleic control. *n* = 6 biological replicates.

**Supplementary Table 1**


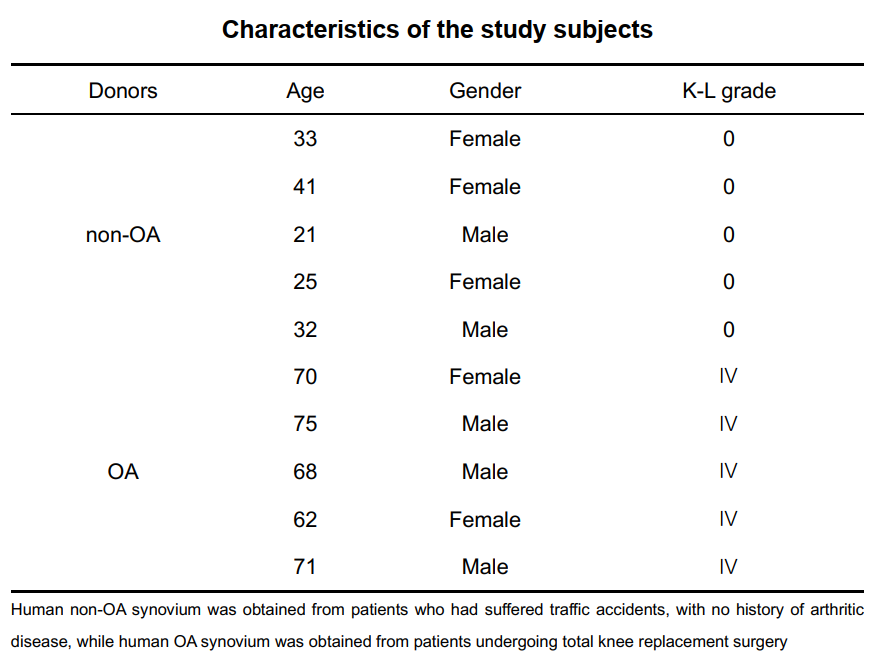

Supplement: Supplementary file 3 — Supplementary file3 (DOCX 3542 KB) [file 18_2023_4975_MOESM3_ESM.docx]
